# A novel UCS memory retrieval-extinction procedure to inhibit relapse to drug seeking

**DOI:** 10.1038/ncomms8675

**Published:** 2015-07-14

**Authors:** Yi-xiao Luo, Yan-xue Xue, Jian-feng Liu, Hai-shui Shi, Min Jian, Ying Han, Wei-li Zhu, Yan-ping Bao, Ping Wu, Zeng-bo Ding, Hao-wei Shen, Jie Shi, Yavin Shaham, Lin Lu

**Affiliations:** 1 Institute of Mental Health/Peking University Sixth Hospital, Key Laboratory of Mental Health, Peking University, Beijing 100191, China.; 2 National Institute on Drug Dependence, Beijing Key Laboratory of Drug Dependence Research, Peking University, Beijing 100191, China.; 3 Department of Biochemistry and Molecular Biology, Basic Medical College, Hebei Medical University, Shijiazhuang 050017, China.; 4 Intramural Research Program, National Institute on Drug Abuse, National Institutes of Health, Baltimore, Maryland 21224, USA.; 5 Peking-Tsinghua Center for Life Sciences, PKU-IDG/McGovern Institute for Brain Research, Peking University, Beijing 100871, China.

## Abstract

We recently reported that a conditioned stimulus (CS) memory retrieval-extinction procedure decreases reinstatement of cocaine and heroin seeking in rats and heroin craving in humans. Here we show that non-contingent cocaine or methylphenidate injections (UCS retrieval) 1 h before the extinction sessions decreases cocaine-priming-induced reinstatement, spontaneous recovery, and renewal of cocaine seeking in rats. Unlike the CS-based memory retrieval-extinction procedure, the UCS memory retrieval manipulation decreases renewal and reinstatement of cocaine seeking in the presence of cocaine cues that were not present during extinction training and also decreases cocaine seeking when the procedure commences after 28 days of abstinence. The inhibitory effect of the UCS retrieval manipulation on cocaine-priming-induced reinstatement is mediated by regulation of AMPA-receptor endocytosis in the basolateral amygdala. The UCS memory retrieval-extinction procedure has superior relapse prevention characteristics than the CS memory retrieval-extinction procedure and could be a promising method for decreasing relapse in human addicts.

## Abstract (web summary)

Cue-based therapies for treating drug addiction have proven to be only partially effective. Here the authors demonstrate a new memory retrieval based treatment protocol for drug addiction that results in long-lasting inhibition of drug seeking behavior in rodents.

## Graphical abstract

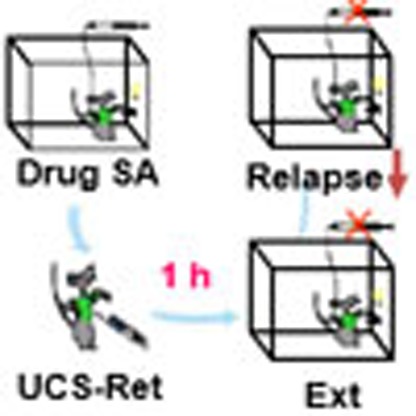

Studies using human addicts or animal models of drug addiction indicate that conditioning factors contribute to drug addiction and that responses to drug-associated cues persist during prolonged abstinence^1^,^2^,^3^,^4^. These findings have led to the development of cue-exposure therapies to extinguish conditioned responses to drug cues^5^. However, cue-exposure therapy in the clinic often fails to prevent relapse when drug addicts return to their home environment^6^. The failure of the cue exposure therapy is likely because of the fact that extinguished responses to conditioned cues can be recovered by acute exposure to the unconditioned stimulus (CS, reinstatement), exposure to the original reward-associated context after extinction of the conditioned responses in a different context (renewal), or passage of time after extinction training (spontaneous recovery)^7^,^8^.

In recent years, investigators have used a CS memory retrieval-extinction procedure to prevent recovery of fear after extinction in rats^9^,^10^ and humans^11^,^12^. In these studies, reinstatement, renewal, and spontaneous recovery of fear responding were decreased by brief exposure to cues previously paired with footshock (a retrieval manipulation) if that exposure was followed 10 min or 1 h later by repeated exposure to the same cues in longer-duration extinction sessions. We recently adapted this memory retrieval-extinction procedure to drug studies in rats (morphine, heroin, and cocaine) and humans (heroin) and found that the procedure decreases reinstatement, spontaneous recovery and renewal (context-induced reinstatement) of drug seeking in rats, and cue-induced drug craving in humans^13^. Our results in rats were independently replicated and extended to drug-induced reinstatement of morphine-conditioned place preference (CPP)^14^,^15^ and renewal of alcohol seeking^16^.

However, there are limitations of the CS memory retrieval-extinction procedure for ‘real world' relapse prevention^17^. First, the inhibitory effect of the procedure is selective to the reactivated cues and does not generalize to other cues that were not reactivated^11^,^12^. Second, inhibition of fear by this procedure only occurs when the memory retrieval-extinction manipulation is performed immediately after fear-conditioning training^10^. Third, for both drug self-administration and CPP, the procedure paradoxically accelerates reacquisition of the previously extinguished conditioned response^15^,^16^.

On the basis of these limitations and results demonstrating that the inhibitory effects of neuropharmacological manipulations on both fear conditioning^18^ and drug CPP^19^,^20^ after memory retrieval was induced by the unconditioned stimulus (UCS), we have recently developed a UCS memory retrieval-extinction procedure and demonstrated its effect on inhibition of Pavlovian conditioned fear in both rats and humans^21^. In the present study, we modified the UCS memory retrieval-extinction procedure to study its effect on reinstatement, spontaneous recovery, and renewal of cocaine seeking in an operant rat model of drug relapse^22^.

We also assessed whether endocytosis of α-amino-3-hydroxy-5-methyl-isoxazole-4-propionic acid (AMPA) receptors in the basolateral amygdala (BLA), which contributes to retrieval and reconsolidation of conditioned fear^10^,^23^,^24^ and methamphetamine^25^ memories, play a role in the inhibitory effect of the UCS memory retrieval manipulation on relapse to cocaine seeking. We also chose to study AMPA receptor endocytosis in BLA because of evidence implicating AMPA receptor trafficking in the nucleus accumbens (NAc) in relapse to cocaine seeking^26^,^27^, and the role of BLA in reconsolidation of memories for cocaine cues and contexts^28^,^29^,^30^.

## Results

In the experiments described below, we first trained all rats to nose poke for intravenous cocaine for 3 h per day for 10–14 days. We then compared the effect of the novel UCS memory retrieval-extinction procedure to our recently established CS memory retrieval-extinction procedure^13^ on reinstatement of cocaine seeking induced by cocaine-priming injections, spontaneous recovery of cocaine seeking after extinction, and renewal (context-induced reinstatement^31^) of cocaine seeking after extinction of cocaine self-administration in a different context. We also assessed the effect of the UCS memory retrieval-extinction procedure on reacquisition of cocaine self-administration under a progressive ratio (PR) schedule. In the different experiments, we have used four to five experimental groups (hereafter termed Group in the statistical analyses) that were exposed to the following manipulations: saline injections 1 h before the extinction sessions, cocaine (3 and/or 10 mg kg^−1^; hereafter termed UCS retrieval) 1 or 9 h before the extinction sessions and 15-min CS retrieval 1 h before the extinction sessions. During the 3-h extinction sessions, active nose-poke responding led to contingent presentations of a tone-light cue previously paired with cocaine injections during training, but cocaine was not delivered. The CS retrieval manipulation was a short 15-min session during which the experimental conditions were identical to those of the extinction sessions^13^. The experimental groups used in each experiment are described in the Methods section and the figures.

The delayed 9-h group (cocaine injections 9 h before the extinction sessions), a standard control condition in studies on memory consolidation^32^ and reconsolidation^33^,^34^,^35^, served as a control condition for the temporal specificity of the UCS memory retrieval-extinction manipulation. We have not included this temporal control condition for the CS retrieval manipulation because we previously found that this manipulation had no effect on cocaine or heroin seeking when it was presented 6 h before the extinction sessions^13^.

### Effect on reinstatement of drug seeking

In Experiment 1, we tested the inhibitory effect of the UCS and CS memory retrieval-extinction procedures on cocaine-priming-induced reinstatement (Supplementary Fig. 1). The statistical analysis included the between-subjects factor of Group and the within-subjects factor of Cocaine priming (0, 5, 10 and 15 mg kg^−1^). We found that exposing rats to the UCS and CS retrieval manipulations 1 h before the extinction sessions decreased cocaine-priming-induced reinstatement of cocaine seeking (*F*_(12,132)_=11.1, *P*<0.01 for Group × Cocaine Priming interaction). No group differences were found for responding on the inactive nose-poke operandum (*P* values>0.1). In addition, both the UCS and CS memory retrieval-extinction manipulations accelerated extinction responding (*F*_(52,572)_=1.8, *P*<0.01 for Group × Extinction Session).

In Experiment 2, we tested the inhibitory effect of the UCS and CS memory retrieval-extinction procedures on spontaneous recovery of cocaine seeking (Supplementary Fig. 2). The statistical analysis included the between-subjects factor of Group and the within-subjects factor of Test session (immediate extinction session, delay 28 day extinction session). We found that exposing rats to the UCS and CS retrieval manipulations 1 h before the extinction sessions decreased spontaneous recovery of cocaine seeking (*F*_(3,36)_=14.5, *P*<0.01 for Group × Test session interaction). No group differences were found for responding on the inactive nose-poke operandum (*P* values>0.1). In addition, both the UCS and CS memory retrieval-extinction manipulations accelerated extinction responding (*F*_(39,468)_=2.0, *P*<0.01 for Group × Extinction Session). We also assessed whether the inhibitory effect of the UCS and CS retrieval manipulations on relapse to cocaine seeking is associated with decreased neuronal activation (c-Fos expression^36^) in medial prefrontal cortex, NAc, and amygdala. Neuronal activity in these brain areas is involved in relapse to drug seeking^37^,^38^,^39^ and conditioned drug effects^40^. We found that both the UCS and CS retrieval manipulations decreased c-Fos expression in the anterior cingulate cortex (ACC), BLA, and NAc core and shell induced by the spontaneous recovery test 28 days after the last extinction session (*F*_(3,23)_=13.7, 9.2, 5.2 and 7.3, *P* values<0.01, for the different brain areas, respectively; Supplementary Fig. 3). In contrast, no group differences in c-Fos expression were found in the central amygdala (CeA), prelimbic (PrL) and infralimbic (IL) medial prefrontal cortex (*P* values>0.1; Supplementary Fig. 4).

In Experiment 3, we tested the inhibitory effect of the UCS and CS memory retrieval-extinction procedures on renewal (context-induced reinstatement) of cocaine seeking (Supplementary Fig. 5). The statistical analysis included the between-subjects factor of Group and the within-subjects factor of Context (last extinction session in context B, test session in context A). We found that exposing rats to the UCS and CS retrieval manipulations 1 h before the extinction sessions decreased renewal of cocaine seeking (*F*_(3,35)_=4.2, *P*<0.05 for Group × Context interaction). No group differences were found for responding on the inactive nose-poke operandum (*P* values>0.1). Additionally, both the UCS and CS memory retrieval-extinction manipulations accelerated extinction responding (*F*_(36,420)_=1.9, *P*<0.01 for Group × Extinction Session). We also found that both the UCS and CS retrieval manipulations decreased c-Fos expression in the ACC, BLA, and NAc core and shell induced by the renewal test 24 h after the last extinction session (*F*_(3,23)_=9.0, 11.2, 4.6 and 8.2, *P* values<0.01, for the different brain areas, respectively, Supplementary Fig. 6). In contrast, no group differences in c-Fos expression were found in CeA, PrL and IL (*P* values>0.1, Supplementary Fig. 7).

### Effect on reacquisition of cocaine self-administration

As mentioned in the Introduction, the CS memory retrieval-extinction procedure paradoxically accelerates reacquisition of the previously extinguished conditioned response for both alcohol self-administration and morphine CPP^15^,^16^. Therefore, in Experiment 4 we tested the effect of the UCS and CS memory retrieval-extinction procedures on reacquisition of cocaine seeking under a PR reinforcement schedule (Supplementary Fig. 8). We chose to assess reacquisition using a PR reinforcement schedule instead of the fixed ratio 1 (FR1) reinforcement schedule we used during self-administration training because the PR reinforcement schedule is thought to provide a more direct measure of the rewarding effects of a drug than continuous reinforcement schedules^41^.

The statistical analysis included the between-subjects factor of Group and the within-subjects factor of Day (the three reacquisition days). We found that exposing rats to the UCS retrieval manipulation (and, to a lesser degree, the CS retrieval manipulation) 1 h before the extinction sessions decreased reacquisition of cocaine self-administration. The statistical analyses showed a main effect of Group for latency to first respond in the first reacquisition session (*F*_(3,34)_=4.9, *P*<0.05), final ratio on the PR schedule *F*_(3,31)_=15.9, *P*<0.01) and total active nose pokes *F*(_3,31_)=11.3, *P*<0.01), but not inactive nose pokes (*P*>0.05). Finally, unlike the results of the previous experiments, the UCS and CS memory retrieval-extinction manipulations did not accelerate extinction responding (*P*>0.1 for Group × Extinction Session).

### Effect on relapse induced by a non-extinguished cocaine cue

**Figure 1:**
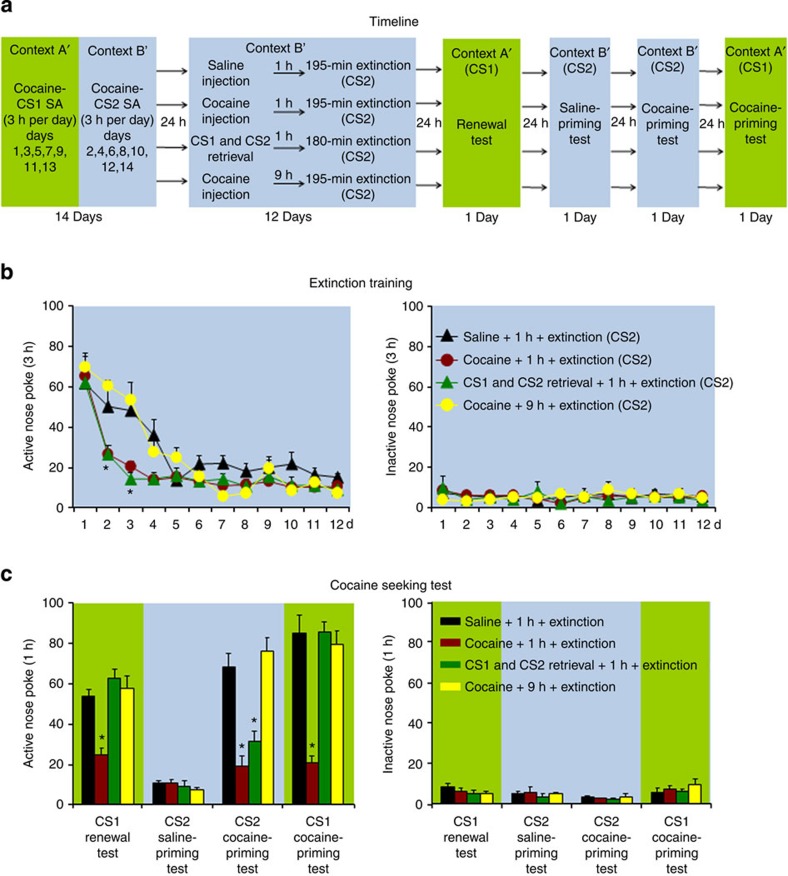
Exposure to the UCS but not the CS memory retrieval-extinction manipulation decreased renewal and cocaine-priming-induced reinstatement when nose-poke responding during testing was reinforced by a non-extinguished discrete CS. (**a**) Timeline of the experimental procedure. (**b**,**c**) The mean number of responses on the active and inactive nose-poke devices during the extinction phase and reinstatement tests.*Different from ‘saline +1 h+extinction', mixed ANOVA, *P<0.05; *n*=7–8 per experimental condition. Error bars represent s.e.m. See Methods and Results for details of the experimental procedure.

In Experiment 5 we assessed whether the inhibitory effect of the UCS and CS memory retrieval-extinction procedures on renewal (context-induced reinstatement) and cocaine-priming-induced reinstatement is also observed when, during the relapse tests, extinguished operant nose-poke responding leads to contingent delivery of a discrete CS cue that had not been previously extinguished during the extinction phase (Fig. 1). As shown in Fig. 1a, we first trained the rats to self-administer cocaine in contexts A′ and B′ where nose pokes for cocaine were paired with CS1 (continuous tone-light cue) or CS2 (intermittent tone-light cue), respectively. Next, we exposed different groups of rats to the UCS and CS retrieval manipulations (both CS1 and CS2 were retrieved in the CS retrieval manipulation) in context B′ before extinction sessions during which nose pokes led to contingent delivery of CS2. Next, we tested the rats in consecutive sessions for renewal in context A′, saline priming in context B′ (baseline vehicle condition) and cocaine priming in contexts A′ and B′ (Fig. 1a).

The main finding in Experiment 5 was that the inhibitory effect of the UCS memory retrieval-extinction manipulation on renewal and cocaine-priming-induced reinstatement was observed for both the extinguished CS (CS2) and the non-extinguished CS (CS1). In contrast, the inhibitory effect of the CS memory retrieval-extinction manipulation was selective to cocaine priming in the presence of the extinguished CS2 in context B′; the CS memory retrieval-extinction manipulation had no effect on either renewal or cocaine-priming-induced reinstatement in context A′ in which nose-poke responding led to contingent delivery of the non-extinguished CS1 during testing (Fig. 1c).

The statistical analysis of nose-poke responding during the renewal test showed a significant effect of Group (*F*_(3,27)_=15.8, *P*<0.01). The analysis of nose-poke responding during the cocaine-priming test in context B′ showed a significant Group × Cocaine priming (saline, 10 mg kg^−1^) interaction (*F*_(3,27)_=21.5, *P*<0.01). The statistical analysis of nose-poke responding during the cocaine-priming test in Context A′ showed a significant effect of Group (*F*_(3,27)_=24.6, *P*<0.01). No group differences were found for responding on the inactive nose-poke operandum in the different tests (*P* values>0.1). In addition, both the UCS and CS memory retrieval-extinction manipulations accelerated extinction responding (*F*_(33,297)_=3.5, *P*<0.01, Fig. 1b).

### Effect on relapse to cocaine seeking after prolonged abstinence

**Figure 2:**
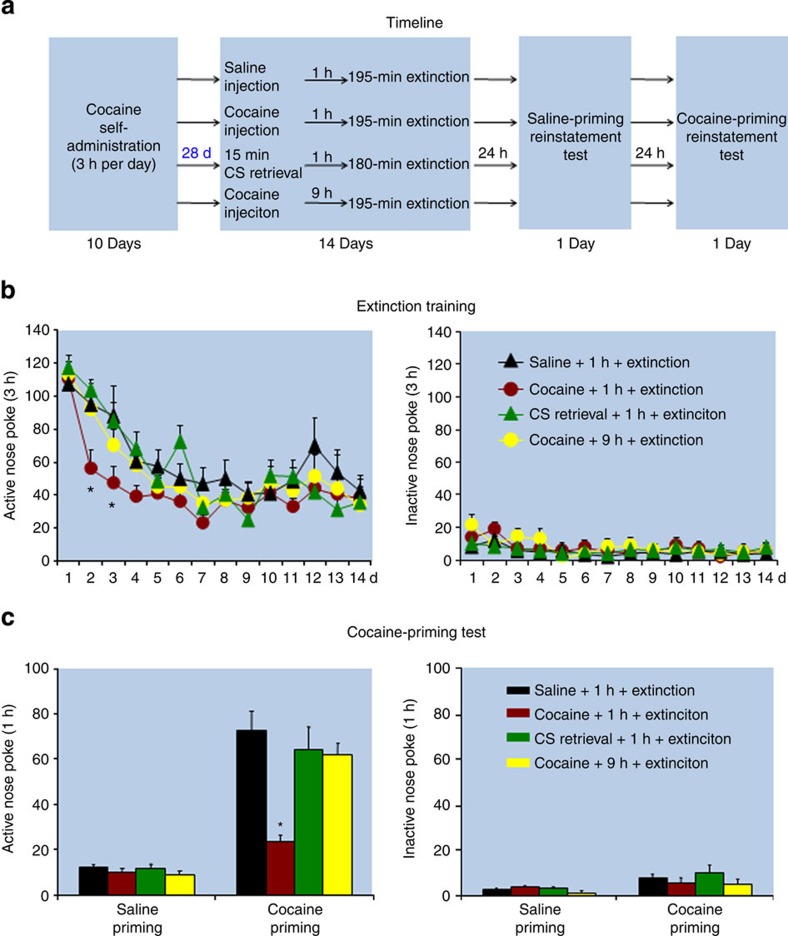
Exposure to the UCS but not the CS memory retrieval-extinction manipulation 28 days after cocaine self-administration training accelerated extinction responding and decreased cocaine-priming-induced reinstatement of drug seeking. (**a**) Timeline of the experimental procedure. (**b**,**c**) The mean number of responses on the active and inactive nose-poke devices during the extinction phase and reinstatement tests. *Different from ‘saline+1 h+ extinction', mixed ANOVA, *P<0.05; *n*=8–9 per experimental condition. Error bars represent s.e.m.

**Figure 3:**
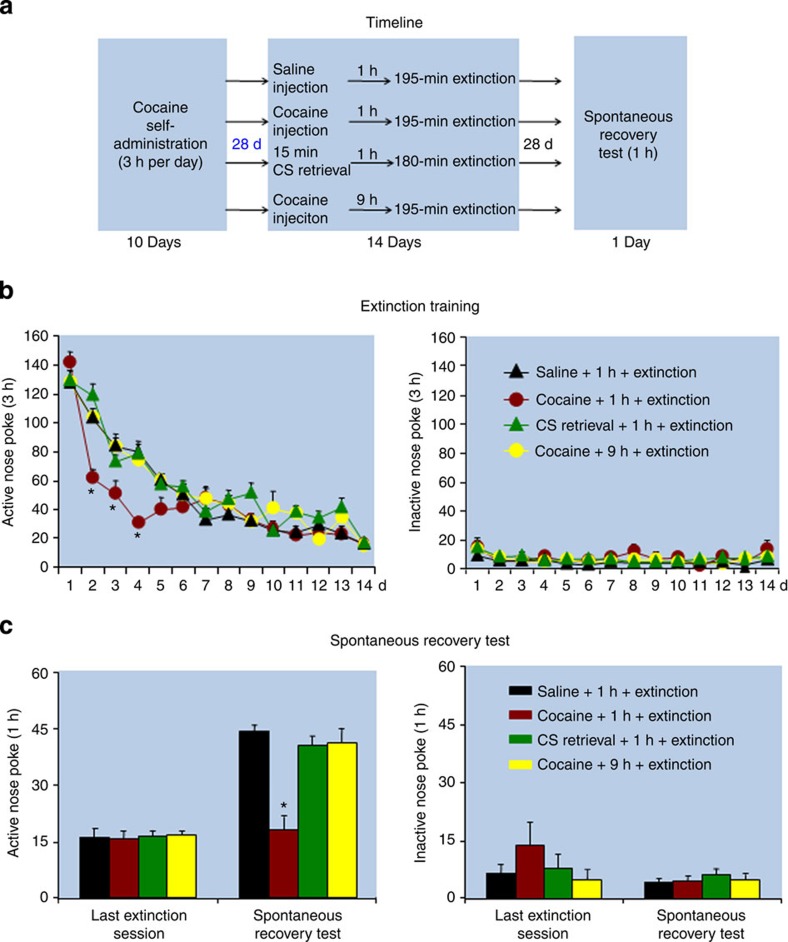
Exposure to the UCS but not the CS memory retrieval-extinction manipulation 28 days after cocaine self-administration training accelerated extinction responding and decreased spontaneous recovery of cocaine seeking. (**a**) Timeline of the experimental procedure. (**b**,**c**) The mean number of responses on the active and inactive nose-poke devices during the extinction phase and spontaneous recovery test. *Different from ‘saline +1 h+extinction', mixed ANOVA, *P<0.05; *n*=11–12 per experimental condition. Error bars represent s.e.m.

**Figure 4:**
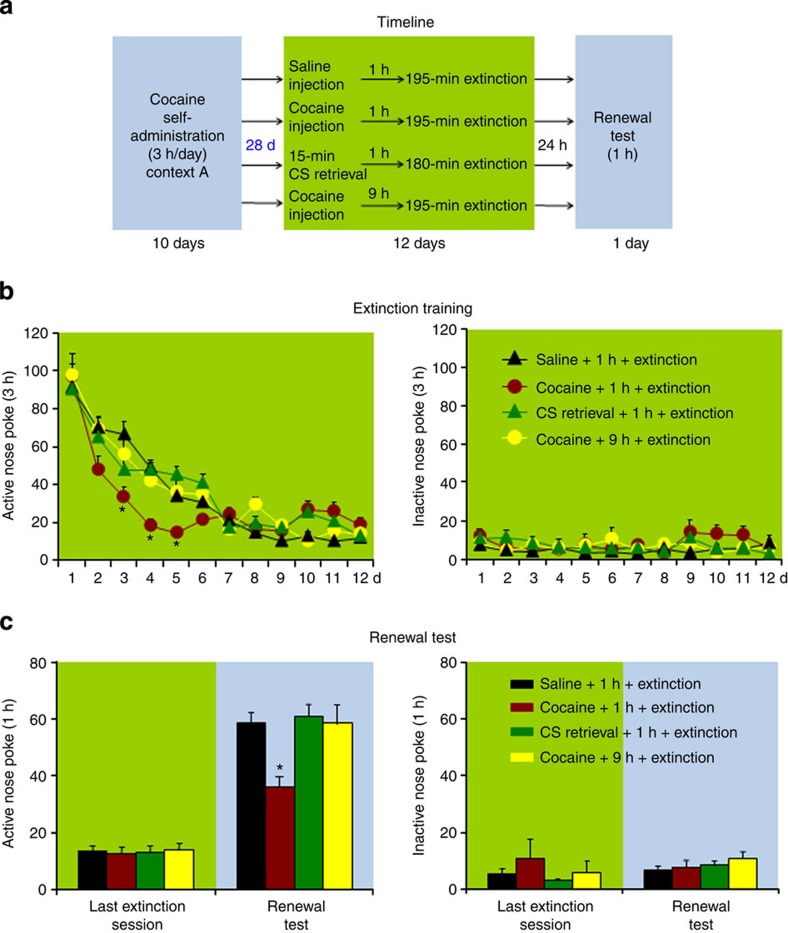
Exposure to the UCS but not to the CS memory retrieval-extinction manipulation 28 days after cocaine self-administration training accelerated extinction responding and decreased renewal of cocaine seeking. (**a**) Timeline of the experimental procedure. (**b**,**c**) The mean number of responses on the active and inactive nose-poke devices during the extinction phase and renewal test. *Different from ‘saline+1 h+extinction', mixed ANOVA, *P<0.05; *n*=11–12 per experimental condition. Error bars represent s.e.m.

Fear-conditioning studies indicate that the inhibitory effect of the CS memory retrieval-extinction procedure on fear inhibition is time-dependent: the procedure is effective when performed immediately after fear-conditioning training but not when it is performed after a delay period between training and the CS retrieval-extinction exposure^10^. On the basis of this finding, in Experiments 6–8 we assessed the effect of the UCS and CS memory retrieval-extinction procedures on reinstatement (cocaine priming), spontaneous recovery, and renewal of cocaine seeking after imposing a 28-day withdrawal (abstinence) period between cocaine self-administration training and the start of the retrieval-extinction procedures. The main finding was that after this withdrawal period, the UCS memory retrieval-extinction manipulation maintained its ability to decrease reinstatement (cocaine priming), spontaneous recovery, and renewal of cocaine seeking, while the CS memory retrieval-extinction manipulation did not (Figs 2, 3, 4).

In Experiment 6 (Fig. 2), we found that exposing rats to the UCS but not the CS retrieval-extinction manipulation 1 h before the extinction sessions decreased cocaine-priming-induced reinstatement of cocaine seeking (*F*_(3,30)_=8.9, *P*<0.01 for Group × Cocaine priming [0, 10 mg/kg] interaction). No group differences were found for responding on the inactive nose-poke operandum (*P* values>0.1). In addition, the UCS but not the CS memory retrieval-extinction manipulations accelerated extinction responding (*F*_(39,390)_=1.5, *P*<0.05 for Group × Extinction session).

In Experiment 7 (Fig. 3), we found that exposing rats to the UCS but not to the CS retrieval-extinction manipulations 1 h before the extinction sessions decreased spontaneous recovery of cocaine seeking (*F*_(3,40)_=13.9, *P*<0.01 for Group × Test session (immediate extinction session, delay 28-day extinction session) interaction). No group differences were found for responding on the inactive nose-poke operandum (*P* values>0.1). In addition, the UCS but not the CS memory retrieval-extinction manipulations accelerated extinction responding (*F*_(39,532)_=4.0, *P*<0.01 for Group × Extinction session).

In Experiment 8 (Fig. 4), we found that exposing rats to the UCS but not the CS retrieval-extinction manipulations 1 h before the extinction sessions decreased renewal of cocaine seeking (*F*_(3,41)_=8.0, *P*<0.01 for Group × Context (last extinction session in context B, test session in context A) interaction). No group differences were found for responding on the inactive nose-poke operandum (*P* values>0.1). In addition, the UCS but not the CS memory retrieval-extinction manipulations, accelerated extinction responding (*F*_(33,451)_=4.2, *P*<0.01 for Group × Extinction session).

### No effect of reversing the UCS retrieval-extinction sequence

In a recent study, Millan *et al*.^16^ trained rats to self-administer alcoholic beer and reported that a short CS memory retrieval (10 min of lever presses that led to contingent presentations of the alcohol-associated discrete cues) that was followed 70 min later by 50 min extinction sessions under the same experimental conditions, decreased renewal (context-induced reinstatement) of alcohol seeking. However, these authors also reported that reversing the experimental sequence (50-min extinction followed 70 min later by 10-min CS retrieval) had the same effect on renewal of alcohol seeking. On the basis of these findings, in Experiment 9 we assessed whether reversing the extinction and the UCS retrieval conditions would affect renewal of cocaine seeking. We found that reversal of the experimental conditions had no effect on renewal of cocaine seeking (Supplementary Fig. 9). The statistical analysis of active nose-poke responding included the between-subjects factor of Group and the within-subjects factor of Context (last extinction session in context B, test session in context A). This analysis showed a significant effect of Context (*F*_(1,15)_=141.1, *P*<0.01) but no significant effects of Group or Group × Context interaction. No group differences were found for responding on the inactive nose-poke operandum (*P* values>0.1). In addition, the reversal manipulation had no effect on extinction responding (*P* values>0.1).

### Effect of inhibition of AMPAR endocytosis in BLA

In Experiments 10–11, we assessed whether endocytosis of AMPA receptors in BLA, which contributes to retrieval and reconsolidation of conditioned fear^10^,^23^,^24^ and methamphetamine^25^ memories, plays a role in the inhibitory effect of the UCS memory retrieval manipulation on relapse to cocaine seeking. As mentioned in the Introduction, we also chose to study AMPA receptor endocytosis in BLA because of evidence implicating AMPA receptor trafficking in the NAc in relapse to cocaine seeking^26^,^27^, and the role of BLA in reconsolidation of memories for cocaine cues and contexts^28^,^29^,^30^.

**Figure 5:**
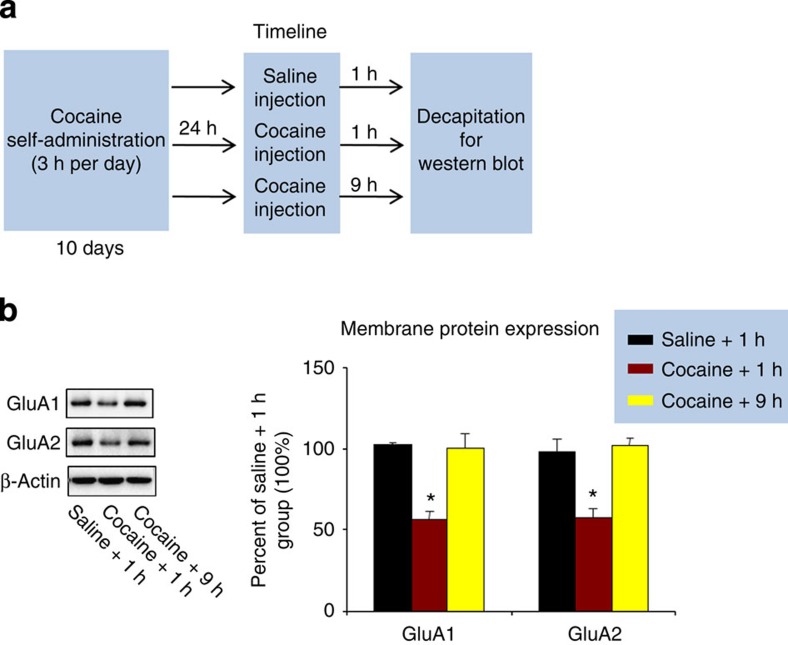
Exposure to UCS decreased membrane expression of GluA1 and GluA2 in BLA. (**a**) Timeline of the experimental procedure. (**b**) Membrane expression of GluA1 and GluA2 proteins after the different experiment manipulations. Values are the mean percentage of protein levels in the BLA in reference to the saline+1 h+extinction group. One-way ANOVA, *Different from group ‘saline+1 h+extinction', *P<0.05; *n*=6 per experimental condition. Error bars represent s.e.m. Uncropped western blots of the data depicted in Fig. 5 are provided in Supplementary Fig. 10.

In Experiment 10 (Fig. 5a), we assessed the effect of UCS retrieval on the membrane expression of GluA1 and GluA2 in BLA. The statistical analysis included the between-subjects factor of Group. We found that membrane levels of GluA1 (*F*_(2,17)_=20.1, *P*<0.01) and GluA2 (*F*_(2,17)_=15.0, *P*<0.01) were decreased 1 h after UCS (non-contingent cocaine) exposure (Fig. 5b and Supplementary Fig. 10).

**Figure 6:**
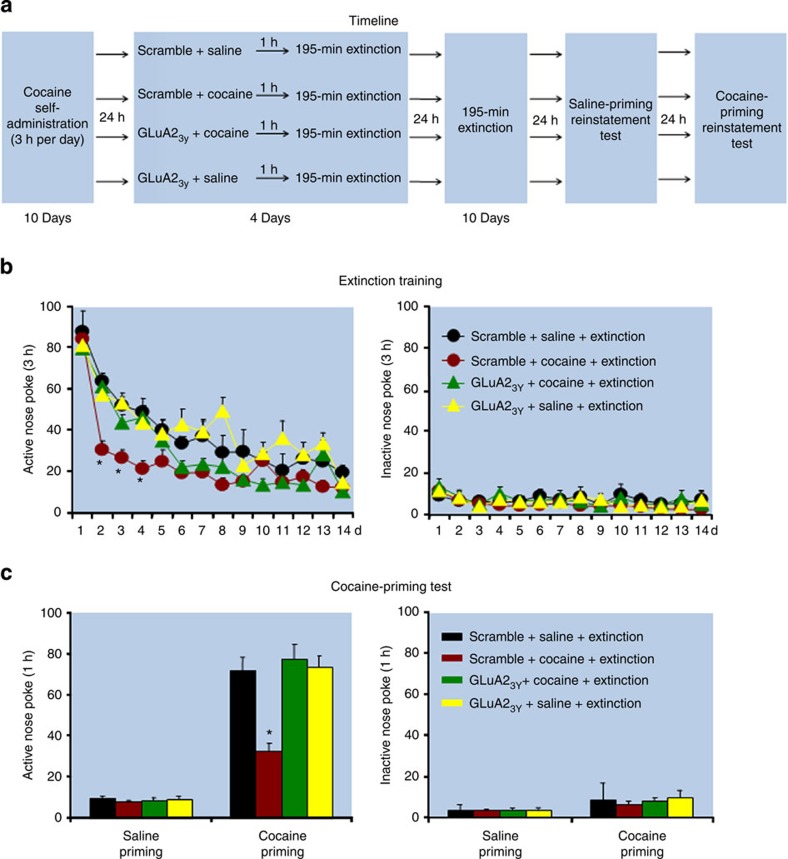
Blockade of endocytosis of AMPAR in BLA inhibits the effect of UCS memory retrieval-extinction procedure on reinstatement of cocaine seeking. (**a**) Timeline of the experimental procedure. (**b,c**) The mean number of responses on the active and inactive nose-poke devices during the extinction phase and the test for cocaine priming (10 mg kg^−1^)-induced reinstatement of cocaine seeking. *Different from other groups, mixed ANOVA, *P<0.05; *n*=9–11 per experimental condition. Error bars represent s.e.m.

In Experiment 11 we assessed whether blockade of endocytosis of AMPAR in BLA would reverse the effect of the UCS memory retrieval-extinction procedure on cocaine-priming-induced reinstatement. For this purpose, we used the GluA2_3y_ peptide, which has been shown in several studies to inhibit GluA2-dependent AMPAR endocytosis^42^,^43^,^44^. We found that injections of GluA2_3y_ into BLA blocked the inhibitory effect of UCS memory retrieval-extinction manipulation on cocaine-priming-induced reinstatement (Fig. 6a; see Supplementary Fig. 11 for the anatomical placement of the intracranial injectors). The statistical analysis, which included the between-subjects factors of UCS Manipulation (saline, cocaine (3 mg kg^−1^)) and GluA2_3y_ Condition (scramble peptide, GluA2_3y_), and the within-subjects factor of Cocaine priming (0, 10 mg kg^−1^), showed a significant interaction between the three factors (*F*_(1,37)_=15.0, *P*<0.01, Fig. 6c). No group differences were found for responding on the inactive nose-poke operandum (*P* values>0.1). In addition, BLA injections of GluA2_3y_ blocked the accelerating effect of UCS memory retrieval-extinction manipulation on extinction responding (*F*_(13,481)_=3.3, *P*<0.01 for UCS Manipulation × GluA2_3y_ Condition × Extinction Session interaction, Fig. 6b).

### Effect of UCS memory retrieval by methylphenidate

**Figure 7:**
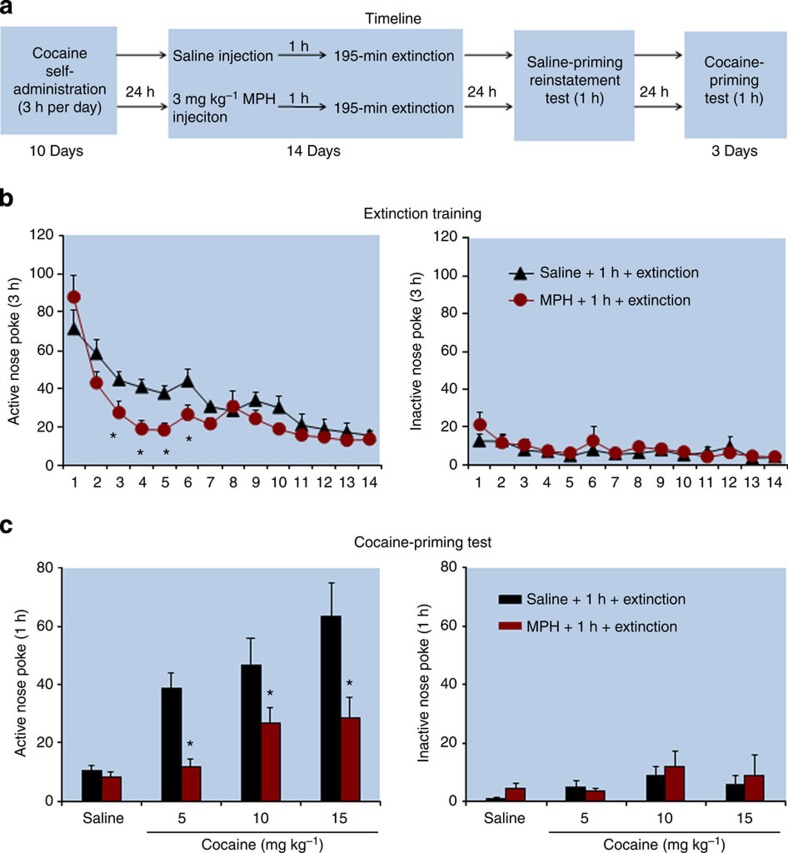
Exposure to methylphenidate 1 h before extinction training accelerated extinction responding and decreased reinstatement of cocaine seeking. (**a**) Timeline of the experimental procedure. (**b,c**) The mean number of responses on the active and inactive nose-poke devices during the extinction phase and the reinstatement test. *Different from ‘saline+1 h+extinction', mixed ANOVA, *P<0.05; *n*=9 per experimental condition. Error bars represent s.e.m. MPH, methylphenidate.

**Figure 8:**
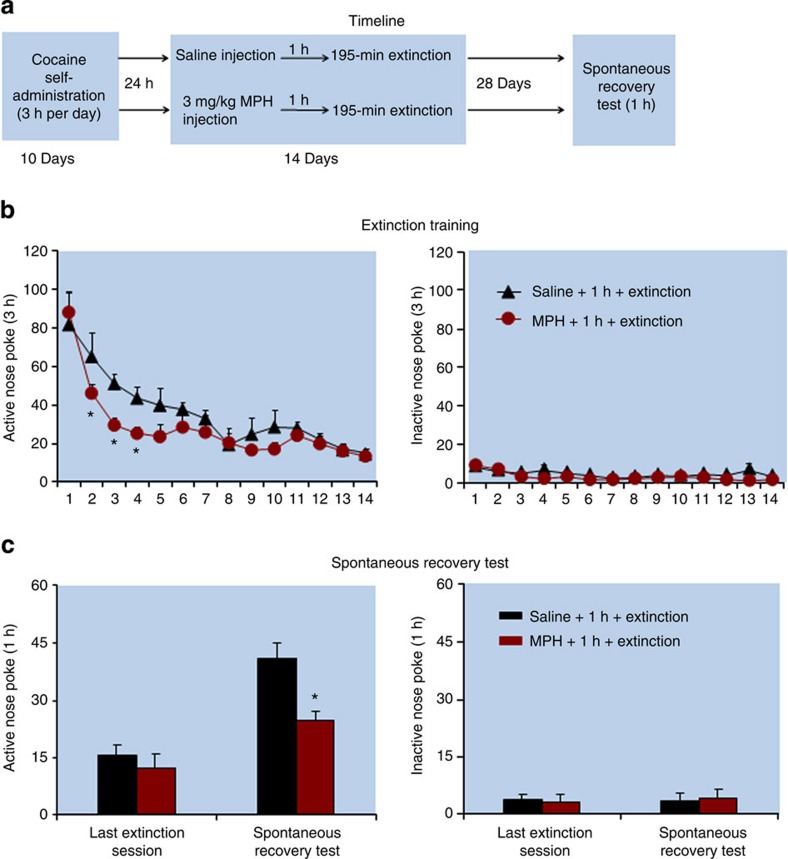
Exposure to methylphenidate 1 h before extinction training accelerated extinction responding and decreased spontaneous recovery of cocaine seeking. (**a**) Timeline of the experimental procedure. (**b,c**) The mean number of responses on the active and inactive nose-poke devices during the extinction phase and spontaneous recovery test. *Different from ‘saline+1 h+extinction', mixed ANOVA, *P<0.05; *n*=9 per experimental condition. Error bars represent s.e.m. MPH, methylphenidate.

Our data indicate that the UCS retrieval procedure using cocaine as the UCS inhibits relapse to cocaine seeking under a wide range of experimental conditions. However, from a treatment perspective, it is highly likely that both clinicians and regulatory agencies would be reluctant to expose cocaine addicts to their abused drug in the clinic. Therefore, in Experiments 12–13 we used four groups of rats to determine the generality of the UCS retrieval manipulation to methylphenidate, an FDA-approved medication. Methylphenidate is a central nervous system stimulant that has been widely used in the treatment of attention deficit hyperactivity disorder^45^. Similar to cocaine, methylphenidate blocks the dopamine transporter, leading to increased concentrations of dopamine in the striatum^46^. Thus, we assessed the effect of exposure to methylphenidate (3 mg kg^−1^, intraperitoneal (i.p.)) 1 h before each extinction session on reinstatement (cocaine priming) and spontaneous recovery. The main finding was that, similar to cocaine, exposure to methylphenidate accelerated extinction responding, and decreased reinstatement (cocaine priming) and spontaneous recovery (Figs 7, 8).

In Experiment 12 (Fig. 7), the statistical analysis, which included the between-subjects factor of Group and the within-subjects factor of Cocaine priming (0, 5, 10 and 15 mg kg^−1^), showed main effects of Cocaine Priming (*F*_(3,48)_=11.4, *P*<0.01) and Group (*F*_(1,16)_=24.0, *P*<0.01). No group differences were found for responding on the inactive nose-poke operandum (*P* values>0.1). In addition, methylphenidate injections accelerated extinction responding (*F*_(13,208)_=2.3, *P*<0.01 for Group × Extinction Session).

In Experiment 13 (Fig. 8), the statistical analysis, which included the between-subjects factor of Group and the within-subjects factor of Test condition (immediate extinction session, delay 28-day extinction session), showed an interaction between the two factors (*F*_(1,16)_=7.8, *P*<0.05). No group differences were found for responding on the inactive nose-poke operandum (*P* values>0.1). In addition, methylphenidate injections accelerated extinction responding (*F*_(13,208)_=24.3, *P*<0.01 for the effect of Extinction Session; *F*_(1,16)_=4.9, *P*<0.05 for group effect).

## Discussion

We developed a UCS memory retrieval-extinction procedure and compared its effects on relapse with the CS memory retrieval-extinction procedure^13^. When the memory retrieval-extinction manipulations started 1 day after cocaine self-administration, both procedures decreased reinstatement (cocaine priming), spontaneous recovery, and renewal (context-induced reinstatement) of cocaine seeking. The UCS retrieval manipulation and, to a lesser degree, the CS retrieval manipulation also decreased the reacquisition of cocaine self-administration. However, when we imposed a 28-day withdrawal period before starting the UCS or CS memory retrieval-extinction procedures, the UCS retrieval manipulation effectively decreased reinstatement, spontaneous recovery and renewal, while the CS retrieval manipulation did not. In addition, the UCS but not CS retrieval manipulation decreased reinstatement and renewal of cocaine seeking in the presence of discrete drug-associated cues that were not previously extinguished during extinction training. Importantly, the inhibitory effect of the UCS memory retrieval manipulation on relapse to cocaine seeking was also observed with methylphenidate, an FDA-approved drug for the treatment of attention-deficit hyperactivity disorder (ADHD). We also found that prior exposure to both the CS and UCS memory retrieval procedures decreased neuronal activation in ACC, BLA and NAc core and shell during the spontaneous recovery and renewal tests. More importantly, our data indicate that endocytosis of AMPAR in BLA plays a critical role in the inhibitory effect of UCS memory retrieval-extinction procedure on reinstatement of cocaine seeking.

Our rationale for the development of the UCS memory retrieval-extinction procedure was based on several recent findings from fear conditioning and drug addiction studies demonstrating potential limitations of the CS memory retrieval-extinction procedure^17^,^47^. These include selectivity of the inhibitory effect of the CS retrieval manipulation to fear^11^,^12^- or drug (present study)-reactivated cues, which does not generalize to other fear or drug cues that were not reactivated. In addition, inhibition of fear or cocaine seeking only occurs when the CS retrieval-extinction manipulation is performed immediately after fear conditioning^10^,^48^ or cocaine self-administration (present study) training. Furthermore, for both alcohol self-administration and morphine CPP, the procedure accelerates reacquisition^15^,^16^; however, we did not replicate these previous findings for cocaine self-administration in the present study. These different results may be because of the use of different animal models (oral alcohol self-administration and morphine CPP versus intravenous cocaine self-administration) and different procedures (extinction in the training context versus extinction in a novel context and assessment of reacquisition using fixed ratio reinforcement schedule versus PR reinforcement schedule).

On the basis of the above limitations of the CS retrieval manipulation, and results demonstrating the inhibitory effects of neuropharmacological manipulations on both fear conditioning^18^ and morphine or cocaine CPP^19^,^20^ after memory retrieval was induced by the UCS (footshock or drug), we have developed the present UCS memory retrieval-extinction procedure. Our results indicate that the range of conditions under which cocaine seeking is inhibited by this procedure was larger than those under which the inhibitory effects of the CS memory retrieval-extinction procedure are observed. Specifically, unlike the CS retrieval manipulation, the UCS retrieval manipulation decreased renewal and reinstatement of cocaine seeking in the presence of cues that were not present during extinction training, and also inhibited cocaine seeking when the procedure started after 28 withdrawal days. In addition, the UCS retrieval manipulation was somewhat more effective than the CS retrieval manipulation in decreasing reacquisition of cocaine self-administration (Supplementary Fig. 8).

The development of the CS memory retrieval-extinction procedure for both fear conditioning^9^,^11^ and drug relapse^13^ was on the basis of theoretical accounts of memory retrieval and reconsolidation and studies on the effects of neuropharmacological manipulations (for example, inhibition of protein synthesis) on reconsolidation of appetitive and aversive memories^34^,^35^,^49^. Reconsolidation refers to a process wherein memories are maintained after their retrieval and destabilization (destabilization refers to the return of a memory to a labile phase after memory retrieval)^47^,^49^. Investigators have inferred that memory reconsolidation was disrupted when post-retrieval neuropharmacological manipulations within a specific time interval (up to 2 h post retrieval, a ‘reconsolidation window') disrupt the expression of the conditioned responses^33^,^35^,^50^.

Within this framework, and the finding that inhibition of protein synthesis in BLA after UCS (shock) retrieval prevented conditioned fear^18^, the finding that our UCS retrieval manipulation decreased reinstatement, spontaneous recovery, and renewal when rats were exposed to non-contingent cocaine injections 1 h but not 9 h before the extinction sessions supports a reconsolidation interpretation of our data. Additional support for the reconsolidation interpretation is that reversal of the experimental conditions—extinction sessions before cocaine UCS exposure—had no effect on renewal of cocaine seeking.

However, a reconsolidation account of our data should be made with caution. First, in most experiments (see figures), nose-poke responding was significantly lower during the last extinction session than during the reinstatement, spontaneous recovery, and renewal tests. Furthermore, the effect of the UCS retrieval manipulation on extinction responding—a behaviour induced by exposure to drug-associated cues^51^—was modest and somewhat inconsistent across experiments (see figures). Therefore, as with the CS retrieval manipulation (present results and ref. 13), the UCS retrieval manipulation only weakened cocaine cue memories (or alternatively decreased the motivational effects of the cues) rather than completely preventing the expression of the conditioned response, as would have been predicted by a reconsolidation account of the data.

One alternative interpretation is that UCS (cocaine) exposure within the consolidation window of extinction memory has strengthened this memory, rendering the original appetitive memory less susceptible to reinstatement, spontaneous recovery, or renewal. In this regard, pharmacological manipulations that promote consolidation of extinction memory decrease reinstatement, spontaneous recovery, and renewal of conditioned fear^52^,^53^. However, an enhancement of extinction consolidation account of our data is somewhat unlikely because cocaine (UCS) exposure 1 h after extinction training (within a putative extinction consolidation window) had no effect on renewal of cocaine seeking.

Another alternative interpretation of the data is that the UCS retrieval manipulation decreased non-reinforced cocaine seeking because repeated non-contingent cocaine exposure before the extinction sessions facilitates discrimination between reinforced and non-reinforced sessions. This notion is based on the account that McNally and colleagues^16^ provided for the mechanism underlying the CS retrieval manipulation to explain their findings that this procedure inhibited renewal of alcohol seeking, but unexpectedly increased reacquisition of alcohol self-administration. However, it is unlikely that the ‘improved discrimination' hypothesis can explain our data because we found that the UCS retrieval manipulation decreased both non-reinforced cocaine seeking during the relapse tests and reacquisition of cocaine self-administration.

Mihindou *et al*.^54^ reported that the effect of cocaine priming (15 mg kg^−1^) on reinstatement after extinction is decreased over repeated testing in the drug context, an effect that is context-dependent. These results may suggest that the inhibitory effect of the UCS retrieval manipulation is merely due to extinction of the response to cocaine priming. This possibility is unlikely for two reasons. First, cocaine has a short half-life in rats (30 min)^55^; thus, it is unlikely that extinction training performed 1 h after injections of a low cocaine dose (3 mg kg^−1^, i.p) was in the presence of pharmacologically relevant doses of cocaine. Second, unlike the finding of Mihindou *et al*.^54^ on the context-dependent effect of their cocaine-priming manipulation, the inhibitory effect of the UCS retrieval manipulation for cocaine priming was context-independent (Fig. 1).

Finally, an important question is the brain mechanisms underlying the inhibitory effect of the UCS retrieval manipulation on cocaine seeking. Our c-Fos data (Supplementary Figs 3 and 6) suggest a role of ACC, BLA, and NAc; brain areas involved in relapse to drug seeking^37^,^38^ and conditioned drug effects^40^. However, from a mechanistic perspective these correlational data should be interpreted with caution because it is unknown whether increased c-Fos expression is the cause or the consequence of the rats' behaviour in the relapse tests. Our data from subsequent experiments, however, demonstrate an important role of AMPAR endocytosis in BLA in the inhibitory effect of the UCS retrieval manipulation on reinstatement of cocaine seeking. Thus, non-contingent cocaine injections (the UCS retrieval manipulation) decreased the expression of GluA1 and GluA2 in the membrane fraction of BLA neurons. More importantly, inhibition of endocytosis of AMPAR by BLA injections of GluA2_3y_ (refs 43, 44) blocked the effect of the UCS memory retrieval-extinction procedure on reinstatement of cocaine seeking. This mechanistic account of the UCS memory retrieval procedure extends findings from previous studies on the role of AMPAR endocytosis in BLA in memory retrieval and reconsolidation^10^,^23^,^24^. However, we cannot exclude that glutamate receptor transmission and synaptic plasticity in other brain regions such as the NAc^26^,^56^,^57^ also plays a role in the inhibitory effect of the UCS retrieval manipulation on cocaine seeking.

Finally, in a recent study we reported on the development of a CS memory retrieval-extinction procedure and demonstrated its efficacy in decreasing drug relapse in a rat model and heroin craving in humans^13^. However, as discussed above and demonstrated in the present report, two main limitations of the CS-based retrieval procedure is that it is not effective after prolonged withdrawal periods and its inhibitory effect on relapse is limited to the reactivated CS. Here we showed that the UCS memory retrieval-extinction procedure can overcome these two main limitations of the CS-based procedure and, importantly, that an FDA-approved drug, methylphenidate, can mimic cocaine's effects in the ‘improved' UCS-based procedure. We propose that using this procedure in the clinic with methylphenidate or other FDA-approved reuptake blockers of the dopamine transporter could be a promising method for decreasing relapse in cocaine addicts.

## Methods

### Subjects

We received male Sprague–Dawley rats, weighing 260–280 g, from the Laboratory Animal Center, Peking University Health Science Center. We housed the rats in groups of five in a temperature- (23±2 °C) and humidity (50±5%)-controlled animal facility with free access to food and water and kept them on a reverse 12-h/12-h light/dark cycle. We performed the experimental procedures in accordance with the National Institutes of Health Guide for the Care and Use of Laboratory Animals and the procedures were approved by the Biomedical Ethics Committee for animal use and protection of Peking University.

### Surgery (Experiments 1–13)

We anaesthetized the rats (weighing 300–320 g when surgery began) with sodium pentobarbital anaesthesia (60 mg kg^−1^, i.p.) and inserted catheters into the right jugular vein with the tip terminating at the opening of the right atrium as previously described^13^,^58^. We allowed the rats to recover for 5–7 days after surgery.

We implanted 23-gauge guide cannulas (Plastics One) 1 mm above the BLA. The coordinates for the BLA were anterior/posterior −2.9 mm, medial/lateral±5.0 mm, dorsal/ventral −8.5 mm (refs 59, 60; Supplementary Fig. 11). We anchored the cannulas to the skull with stainless steel screws and dental cement. We inserted a stainless steel blocker into each cannula to maintain patency and prevent infection.

### Intravenous cocaine self-administration training (Experiments 1–13)

The chambers (AniLab Software & Instruments) were equipped with two nose-poke operandi (AniLab Software & Instruments) located 5 cm above the floor of the chambers. Nose pokes in one (active) operandum led to cocaine infusions that were accompanied by a 5-s tone-light cue. Nose pokes in the other (inactive) operandum were also recorded but had no consequence. We trained rats to self-administer cocaine hydrochloride (0.75 mg kg^−1^ per infusion) during three 1-h daily sessions (separated by 5 min) over 10 days. The sessions began at the onset of the dark cycle. We used an FR1 reinforcement schedule with a 40-s timeout period after each infusion. For reacquisition of cocaine self-administration, we used a progressive-ratio reinforcement schedule in which the requirements after each reward delivery were increased in the following way: 1, 2, 4, 6, 9, 12, 15, 20 and so on, as described previously^41^.

Each session began with the illumination of a house light that remained on for the entire session. We limited the number of cocaine infusions to 20 h^−1^ to prevent overdose. At the end of the training phase, we matched the groups in the different experimental conditions for their cocaine intake during training. We used these training conditions on the basis of our previous study^13^.

### Retrieval trials induced by UCS or CS

*UCS retrieval manipulation*: We injected the rats non-contingently with saline (0.5 ml, i.p.) or the previously self-administered drug (UC; cocaine (3 or 10 mg kg^−1^, i.p.)) in their home cage at different time points before the start of the 12- to 14-daily 195-min extinction sessions. We based the cocaine UCS retrieval doses on previous studies using the reinstatement procedure^51^,^61^; these studies also guided our choice for the cocaine-priming doses during the reinstatement tests described below.

*CS retrieval manipulation:* This manipulation was identical to the one used in our previous study^13^. We gave the rats short (15 min) daily sessions during which nose-poke responding led to contingent delivery of the 5-s tone-light cue but not cocaine. We started the 180-min daily extinction sessions 1 h after the CS retrieval manipulation.

### Extinction of drug-reinforced responding

During the extinction sessions (195 min for the UCS retrieval condition and 180 min for the CS retrieval condition), the conditions were the same as during training, except that active nose-poke response resulted in the delivery of the tone-light cue, but not cocaine infusions. We gave the rats 12–14 daily extinction sessions.

### Test for reinstatement of drug seeking

The test conditions were the same as during training (and the CS retrieval manipulation, except for Experiment 5), with the exception that active nose pokes were not reinforced by cocaine. Each session began with illumination of the house light, which remained on for the entire session. Nose-poke responding during the test sessions resulted in contingent presentations of the tone-light cue that had previously been paired with drug infusions but not cocaine. During the drug-priming reinstatement tests, we injected cocaine 5 min before the start of the sessions.

### Immunohistochemistry

We performed immunohistochemistry for c-Fos in brain tissue sections according to previous studies with minor modifications^62^,^63^. Thirty minutes after the behavioural tests, we perfused six randomly chosen rats per group with 4% paraformaldehyde, removed the brains and post-fixed them for 24 h. We then sectioned the brains (30-μ coronal sections) with a microtome. We collected every third serial section on gelatin-coated microscope slides. We then placed the sections in freshly prepared methanol–H_2_O_2_ solution for 10 min to block endogenous peroxidase activity. After incubation with rabbit anti-Fos (sc-52, Lot #J2313, Santa Cruz Biotechnology; 1:200 dilution in PBS, overnight at 4 °C, then 30 min at 37 °C), the tissue sections were washed three times in PBS, followed by additional 1 h incubation with biotin-conjugated second antibody and three washes with PBS. We then incubated the sections for 10 min in streptavidin peroxidase and washed them three times in PBS. Next, we reacted the sections with a 0.05% solution of 3,30-diaminobenzidine (DAB, Beijing Zhongshan Golden-Bridge Biological Technology) and 0.01% H_2_O_2_ in 0.1-M PBS. Incubation times varied from 3 to 10 min, depending on the expression levels of the DAB reaction product determined using microscopy.

To exclude the possibility that different developmental procedures across treatment groups would confound the Fos comparisons we incubated sections from a given brain area from the different groups for an equal time. We assessed Fos protein expression in the ACC, PrL, IL, CeA, BLA, and NAc core and shell. The number of Fos-positive cells in these brain regions was counted according to previous studies from our laboratory^64^,^65^, in which two or three sections from each brain region for each rat were selected. The cell numbers on either side of a given brain region were averaged and taken as the positive immunoreactive cell number for each rat. We measured the number of Fos-labelled cells using a cast-grid microscope (MetaMorph/DP10/Bx41, UIC/Olympus, US/JP) with an image-analysis programme (MetaMorph, version 4.65).

### Intracranial injections

We injected Tat-GluA2_3Y_ (45 pmol per side) and Scrambled Tat-GluA2_3Y_ (45 pmol per side) bilaterally into BLA with Hamilton syringes connected to 30-gauge injectors (Plastics One). We based the dose of the peptides on previous reports^44^,^66^. We injected a total volume of 0.5 μl bilaterally over 1 min and kept the injector in place for an additional 1 min to allow for diffusion. At the end of the experiments, we anaesthetized the rats with sodium pentobarbital (100 mg kg^−1^, i.p.) and transcardially perfused them. We verified cannula placements using Nissl staining with a section thickness of 40 μm under light microscopy. We excluded three rats with misplaced cannulas from the statistical analysis.

### Western blot assays

We based the assay's procedures on those used in our previous studies^21^,^67^,^68^. After decapitation, we rapidly extracted the rats' brains, froze them in −60 °C N-hexane and then transferred the brains to −80 °C freezer. We placed bilateral tissue punches (12 gauge) of the BLA in a 1.5-ml microtube that contained ice-cold homogenization buffer (0.32 M sucrose, 4 mM HEPES, 1 mM EDTA, 1 mM EGTA and protease/phosphatase inhibitor cocktail, pH 7.4). After homogenizing the sample by an electrical disperser (Wiggenhauser, Sdn Bhd), we centrifuge the homogenate at 1,000*g* for 10 min at 4 °C to obtain the pellet (P1) that contained nuclei and large debris. We centrifuged again the supernatant (S1) at 10,000*g* for 30 min at 4 °C to generate a crude synaptosomal fraction (P2) and supernatant (S2; the cytosolic fraction). We lysed the crude synaptosomal membrane pellet (P2) hypo-osmotically and centrifuged it at 25,000*g* for 30 min at 4 °C to generate the synaptosomal membrane fraction (LP1). The S2 and LP1 were separately resuspended in HEPES-lysis buffer (50 mM HEPES, 1 mM EDTA, 1 mM EGTA and protease/phosphatase inhibitor cocktail, pH 7.4). We determined the protein concentrations of all of the samples (S2 and LP1) using the bicinchoninic acid assay (Beyotime Biotechnology).

We further diluted the samples in HEPES-lysis buffer to equalize the protein concentrations. We added 4 × loading buffer (16% glycerol, 20%-mercaptoethanol, 2% SDS and 0.05% bromophenol blue) was added to each sample (3:1, sample:loading buffer) before boiling for 3 min. We cooled the samples and subjected them to SDS–polyacrylamide gel electrophoresis (10% acrylamide/0.27% N,N′-methylenebisacryalamide resolving gel) for ∼40 min at 80 V in stacking gel and ∼1 h at 130 V in resolving gel. For each electrophoresis run, increasing amounts of protein pooled from the brain region being tested were used to produce a standard curve. We transferred the proteins electrophoretically to Immobilon-P transfer membranes (Millipore) at 0.25A for 2.5 h. We washed the membranes with TBST (Tris-Buffered Saline plus 0.05% Tween-20, pH 7.4) and then placed them in blocking buffer (5% bovine serum albumin (BSA in TBST) overnight at 4 °C.

We then incubated the membranes for 1 h at room temperature on an orbital shaker with anti-GluR1 (ab109450, 1:1,000; Abcam), anti-GluR2 (ab52932, 1:1,000; Abcam) or β-actin (sc-47778, 1:1,000; Santa Cruz) in TBST plus 5% BSA and 0.05% sodium azide. After three 5-min washes in TBST buffer, we incubated the blots for 45 min at room temperature on a shaker with horseradish peroxidase-conjugated secondary antibody (goat anti-rabbit IgG; Santa Cruz; PI-1,000; Vector Labs) diluted 1:5,000 in blocking buffer. We then washed the blots three times for 5 min each in TBST and incubated with a layer of Super Signal Enhanced chemiluminescence substrate (Detection Reagents 1 and 2, 1:1 ratio, Pierce Biotechnology) for 1 min at room temperature. We removed excess mixture before the wrapping the blots with a clean piece of plastic wrap (no bubbles between blot and wrap) and detected the blots by ChemiDoc MP (Bio-Rad). We quantified band intensities using two observers who were blind to the experimental groups using the Quantity One software (version 4.4.0, Bio-Rad). We compared band intensities from each test sample to the band intensities from the standard curves. The amount of the protein of interest in each sample was interpolated from the standard curve. The standard curve runs in all western blots in our studies demonstrate that the band intensities for each of our test samples were within the linear range of detection.

### Specific experiments

*Experiment 1: Effect of UCS and CS retrieval manipulations on cocaine-priming-induced reinstatement*: We trained rats to self-administer intravenous cocaine during three 1-h daily sessions over 10 days. After training, we divided the rats into five experimental groups: saline injections 1 h before the extinction sessions, cocaine (3 mg kg^−1^) 1 or 9 h before the extinction sessions, cocaine (10 mg kg^−1^) 1 h before the extinction sessions and 15-min CS retrieval 1 h before the extinction sessions. We subsequently tested all rats for reinstatement of nose-poke responding after priming non-contingent injections of saline (0.5 ml, i.p.) and cocaine (5,10, and 15 mg kg^−1^, i.p.) in counterbalanced order over 4 days.

*Experiment 2: Effect of UCS and CS retrieval manipulations on spontaneous recovery of cocaine seeking* : We trained rats to self-administer intravenous cocaine during three 1-h daily sessions over 10 days. After training, we divided the rats into four experimental groups: saline injections 1 h before the extinction sessions, cocaine (3 mg kg^−1^) 1 or 9 h before the extinction sessions, and 15-min CS retrieval 1 h before the extinction sessions. We subsequently tested all rats for spontaneous recovery 28 days after the last extinction session. Thirty minutes after the spontaneous recovery tests, we deeply anaesthetized the rats, perfused them and extracted their brains for subsequent immunohistochemistry assays of Fos.

*Experiment 3: Effect of UCS and CS retrieval manipulations on spontaneous recovery of cocaine seeking:* We trained rats to self-administer intravenous cocaine during three 1-h daily sessions over 10 days in Context A. After training, we divided the rats into four experimental groups that underwent the UCS and CS retrieval manipulation in Context B: saline injections 1 h before the extinction sessions, cocaine (3 mg kg^−1^) 1 or 9 h before the extinction sessions and 15-min CS retrieval 1 h before the extinction sessions. The experimental procedure is based on previous studies^69^,^70^, and the two counterbalanced contexts differed from each other as follows: context A had smooth stainless steel rod floor and grey walls; context B had granular flat floor and wallpaper with black and white stripes covered walls. Subsequently, we tested all rats for context-induced reinstatement (renewal) of cocaine seeking in Context A. Thirty minutes after the renewal tests we deeply anaesthetized the rats, perfused them and extracted their brains for subsequent immunohistochemistry assays of Fos.

*Experiment 4: Effect of the UCS and CS retrieval manipulations on reacquisition of cocaine self-administration:* We trained rats to self-administer intravenous cocaine during three 1-h daily sessions over 10 days. After training, we divided the rats into four experimental groups: saline injections 1 h before the extinction sessions, cocaine (3 mg kg^−1^) 1 or 9 h before the extinction sessions and 15-min CS retrieval 1 h before the extinction sessions. We subsequently tested all rats for reacquisition of cocaine self-administration for 2 h per day under the PR schedule (see above). We chose to assess reacquisition using this schedule instead of the FR1 reinforcement schedule because PR schedules are thought to provide a more direct measure of the rewarding effects of a drug than continuous reinforcement schedules^41^.

*Experiment 5: Effect of UCS and CS retrieval manipulations on cocaine seeking induced by cocaine cues not present during extinction training:* We trained rats to self-administer intravenous cocaine during three 1-h daily sessions. On training days 1, 3, 5, 7, 9, 11 and 13, we paired cocaine infusions with tone-light cue-1 (continuous 5 s, CS1) in context A′; on training days 2, 4, 6, 8, 10, 12 and 14 we paired cocaine infusions with tone-light cue-2 (intermittent 5 s: 50 ms × 100 with 50-ms interval, CS2) in context B′. Thus, the same tone-light cue was given either continuously or intermittently. The contexts were the same as described in Experiment 3. After training, we divided the rats into four experimental groups that underwent the UCS or CS retrieval manipulations in context B′: saline injections 1 h before the extinction sessions, cocaine (3 mg kg^−1^) 1 or 9 h before the extinction sessions and 15-min CS1 retrieval plus 15-min CS2 retrieval 1 h before the extinction sessions. During the extinction sessions, active nose poke led to contingent presentation of CS2. We then performed four consecutive tests: renewal (context-induced reinstatement) test in which nose pokes were reinforced by CS1 in context A′, saline priming in context B′ (a baseline vehicle condition), cocaine priming (10 mg kg^−1^) in context B′ and cocaine priming in context A′. During the tests in context A′ and context B′, nose pokes led to contingent delivery of CS1 and CS2, respectively.

*Experiment 6: Effect of UCS and CS retrieval manipulations on cocaine-priming-induced reinstatement after 28 withdrawal days:* The experimental procedure for Experiment 6 was identical to that of Experiment 1, except that the retrieval-extinction manipulations started 28 days after training. The experiment included four groups: saline injections 1 h before the extinction sessions, cocaine (3 mg kg^−1^) 1 or 9 h before the extinction sessions and 15-min CS retrieval 1 h before the extinction sessions.

Experiment *7: Effect of UCS and CS retrieval manipulations on spontaneous recovery after 28 withdrawal days:* The experimental procedure and groups for Experiment 7 were identical to that of Experiment 2, except that the retrieval-extinction manipulations started 28 days after training.

*Experiment 8: Effect of UCS and CS retrieval manipulations on renewal after 28 withdrawal days:* The experimental procedure and groups for Experiment 8 were identical to that of Experiment 3, except that the retrieval-extinction manipulations started 28 days after training.

*Experiment 9: Effect of reversed sequence of the UCS-retrieval and extinction on renewal of cocaine seeking:* The experimental procedure for Experiment 9 was identical to that of Experiment 3, except that the UCS retrieval manipulation was given 1 h after the extinction sessions. The experiment included two groups: saline or cocaine injections 1 h after the extinction sessions.

*Experiment 10: Effect of CS or UCS retrieval on endocytosis of GluA1 and GluA2 in BLA:* We trained rats to self-administer intravenous cocaine during three 1-h daily sessions over 10 days. After training, we divided the rats into three experimental groups: saline injections 1 h before the decapitation and cocaine (3 mg kg^−1^) 1 or 9 h before the decapitation. We extracted the BLA of the rats for subsequent western blot assays of GluA1 and GluA2.

*Experiment 11: Effect of injections of GluA2*_*3y*_ *into BLA on the UCS memory retrieval-extinction manipulation:* We trained rats to self-administer intravenous cocaine during three 1-h daily sessions over 10 days. After training, we divided the rats into four experimental groups: injections of scramble GluA2_3y_ into BLA+saline injection (1 ml kg^−1^, i.p.), injections of scramble GluA2_3y_ into BLA followed by cocaine injection (3 mg kg^−1^, i.p.), injections of GluA2_3y_ into BLA followed by saline injection (i.p.), and injections of GluA2_3y_ into BLA followed by cocaine injection (3 mg kg^−1^, i.p.). All rats received extinction training after the different manipulations 1 h later. We subsequently tested all rats for reinstatement of nose-poke responding after priming non-contingent injections of saline (0.5 ml, i.p.) and cocaine (10 mg kg^−1^, i.p.).

*Experiment 12: Effect of exposure to methylphenidate 1 h before extinction training on reinstatement of cocaine seeking:* We trained rats to self-administer intravenous cocaine during three 1-h daily sessions over 10 days. After training, we divided the rats into two experimental groups: saline injections 1 h before the extinction sessions and methylphenidate (3 mg kg^−1^) injections 1 h before the extinction sessions. We subsequently tested all rats for reinstatement of nose-poke responding after priming non-contingent injections of saline (0.5 ml, i.p.) and cocaine (5, 10 and 15 mg kg^−1^, i.p.) in counterbalanced order over 4 days.

*Experiment 13: Effect of exposure to methylphenidate 1 h before extinction training on spontaneous recovery of cocaine seeking:* We trained rats to self-administer intravenous cocaine during three 1-h daily sessions over 10 days. After training, we divided the rats into two experimental groups: saline injections 1 h before the extinction sessions and methylphenidate (3 mg kg^−1^) injections 1 h before the extinction sessions. We subsequently tested all rats for spontaneous recovery 28 days after the last extinction session.

### Statistical analysis

We report the results as mean±s.e.m. and analysed the data by analyses of variance (ANOVAs) with the appropriate between- and within-subjects factors for each experiment (see Results section). We followed up on significant main effects and interactions (*P<*0.05, two-tailed) from the factorial ANOVAs using Turkey's *post hoc* tests.

## Additional information

**How to cite this article:** Luo, Y.-X. *et al*. A novel UCS memory retrieval-extinction procedure to inhibit relapse to drug seeking. *Nat. Commun.* 6:7675 doi: 10.1038/ncomms8675 (2015).

## Supplementary Material

Supplementary Information Supplementary Figures 1-11
