# Supplementary material for: A novel UCS memory retrieval-extinction procedure to inhibit relapse to drug seeking
Source: Nat Commun. 2015 Jul 14;6:7675. doi: 10.1038/ncomms8675 (PMC4510700; doi:10.1038/ncomms8675)
Supplement: Supplementary Information — Supplementary Figures 1-11 [file ncomms8675-s1.pdf]

May 22, 2015
Nature Communications

Supplementary information

A novel UCS memory retrieval-extinction procedure to inhibit relapse to drug seeking

Yi-xiao Luo[1,2,*], Yan-xue Xue[2,*], Jian-feng Liu[1,2], Hai-shui Shi[3], Ming Jian[1,2], Ying Han[1,2], Wei-li Zhu[2], Yan-ping Bao[2], Ping Wu[2], Zeng-bo Ding[2], Hao-wei Shen[2], Jie Shi[2], Yavin Shaham[4], Lin Lu[1,2,5 #]

Supplementary figure 1-11

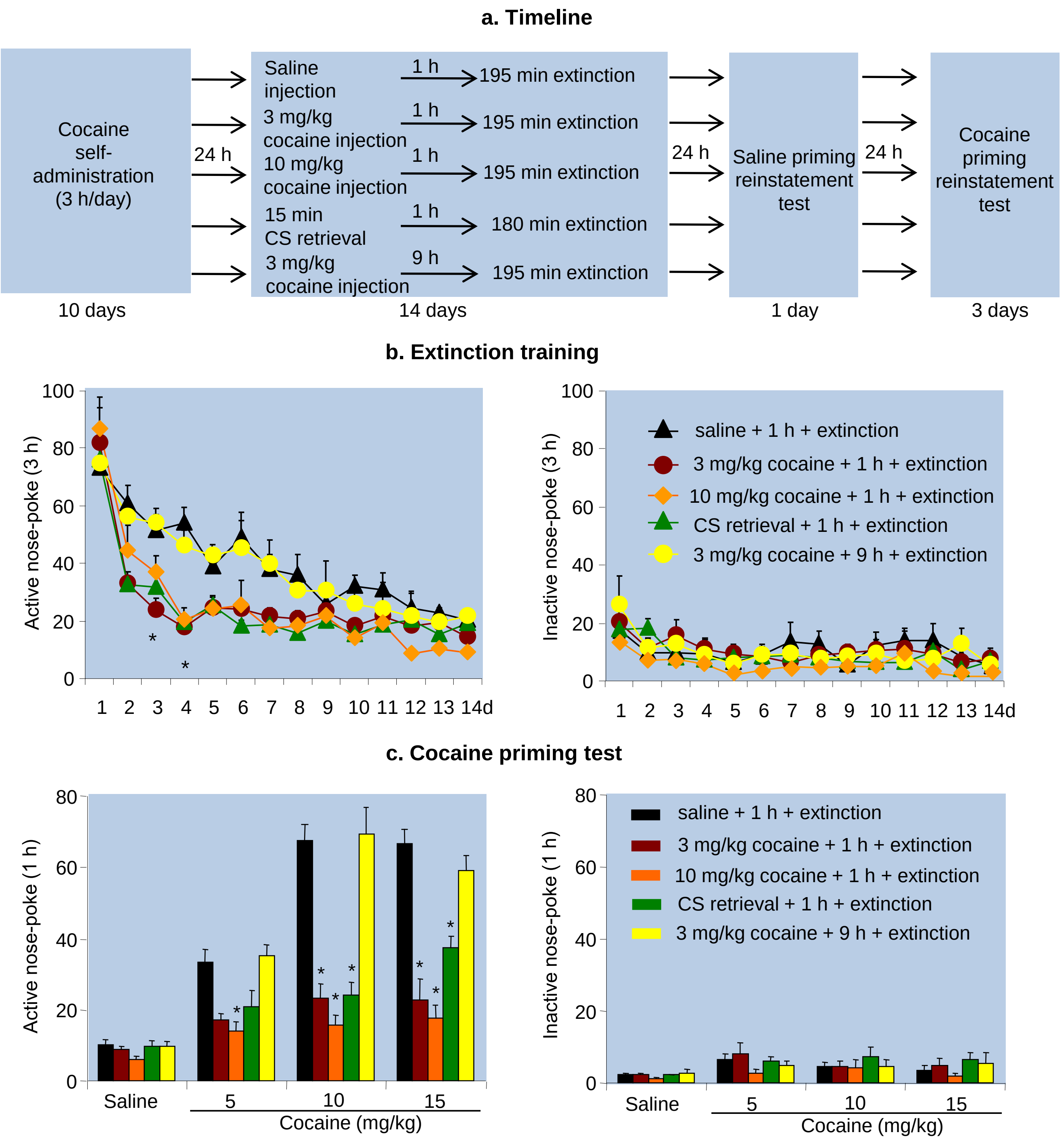

Supplementary Figure 1. *Exposure to the UCS and CS memory retrieval-extinction manipulations accelerated extinction responding and decreased cocaine-priming-induced reinstatement of drug seeking.* (**a**) Timeline of the experimental procedure. (**b-c**) Mean±SEM number of responses on the active and inactive nose-poke devices during the extinction phase and reinstatement test, n=9-10 per experimental condition. * Different from “saline + 1 h + extinction”, mixed ANOVA**,** p<0.05.

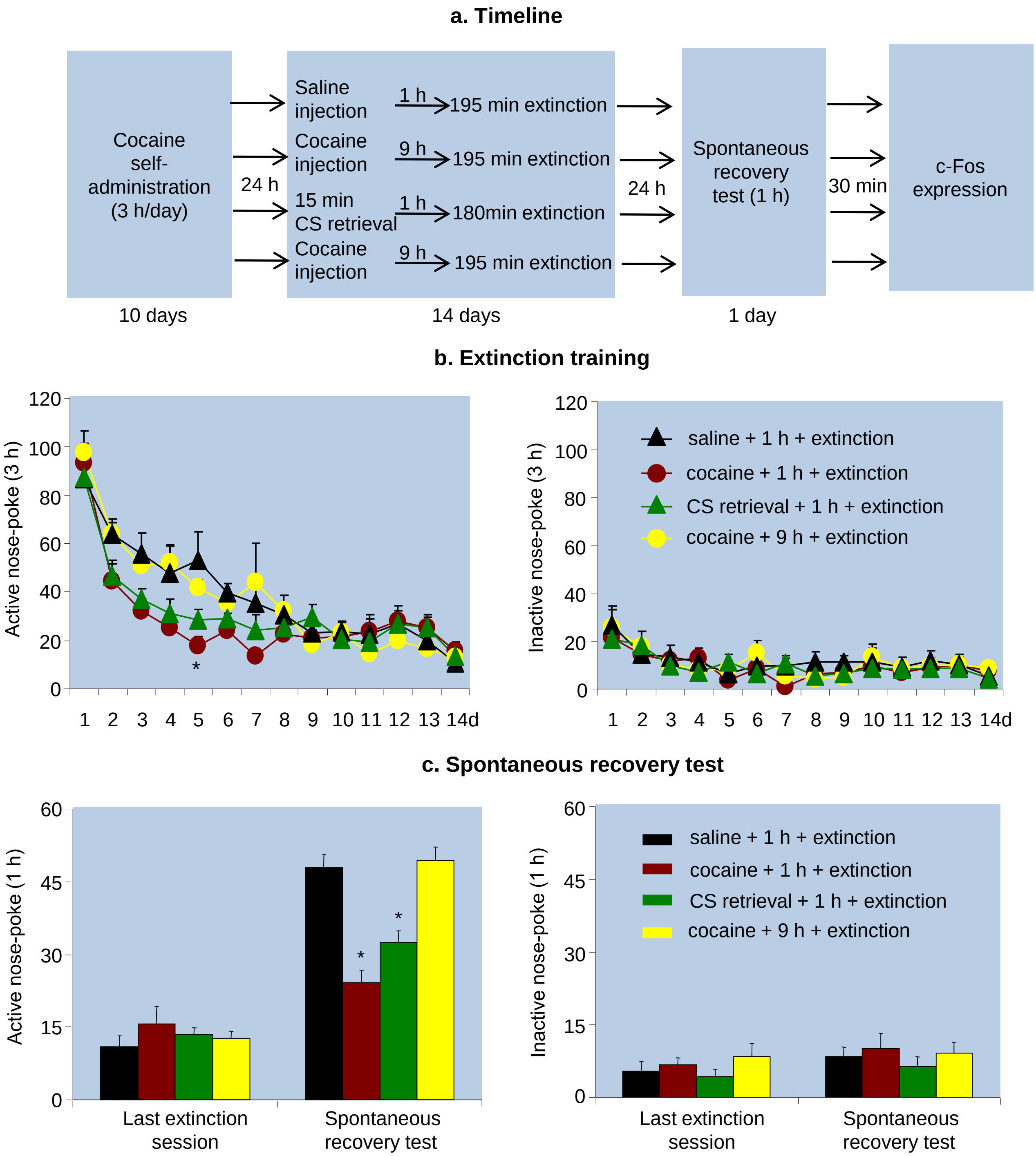

Supplementary Figure 2. *Exposure to the UCS and CS memory retrieval-extinction manipulations accelerated extinction responding and decreased spontaneous recovery of cocaine seeking*. (**a**) Timeline of the experimental procedure. (**b-c**) Mean±SEM number of responses on the active and inactive nose-poke devices during the extinction phase and spontaneous recovery test, n=10 per experimental condition. * Different from “saline + 1 h + extinction”, mixed ANOVA**,** p<0.05.

a. Photomicrograghs of representative images for c-Fos staining at 20X magnificant

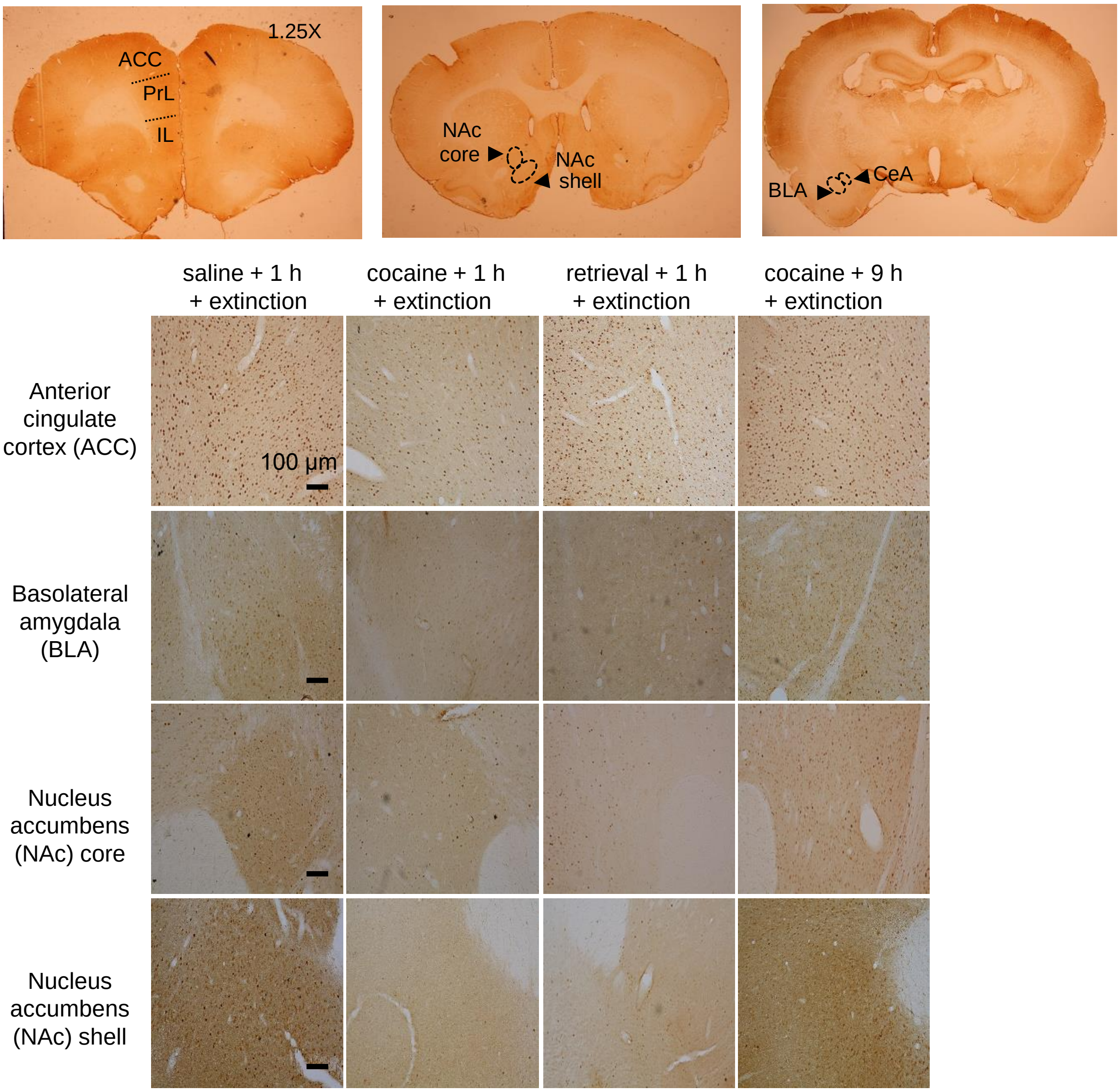

b. Number of c-Fos positive cells

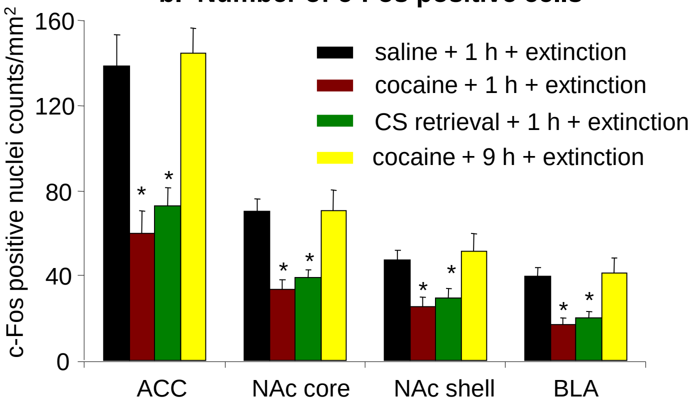

Supplementary Figure 3. *Exposure to the UCS and CS memory retrieval-extinction manipulations decreased c-Fos expression in ACC, NAc core, NAc shell and BLA 30 min after the spontaneous recovery test.* (d) Photomicrograghs of representative images for c-Fos staining at 1.25X and 20X magnification in ACC, BLA, and NAc core and shell. (e) Expression of c-Fos protein after the spontaneous recovery test; values are mean ± SEM number of c-Fos-positive cell counts per mm^2 in the different brain regions, n=6 per experimental condition. * Different from “saline + 1 h + extinction”, one-way ANOVA**,** $p<0.05$.

a. Photomicrograghs of representative images for c-Fos staining

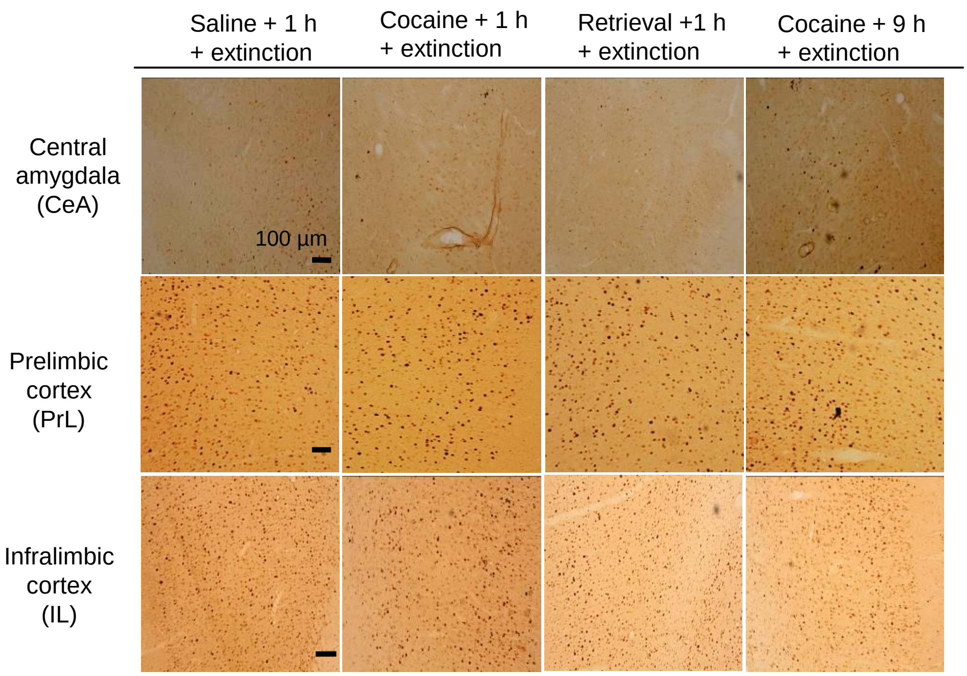

b. Number of c-Fos positive cells

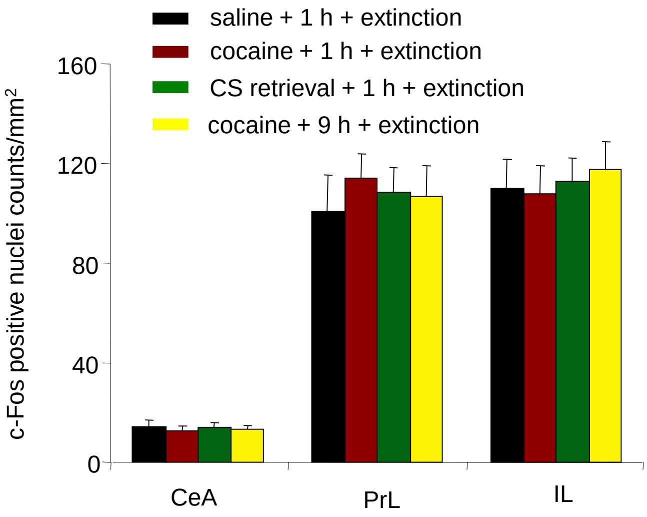

Supplementary Figure 4. *Exposure to the UCS and CS memory retrieval-extinction manipulations had no effect on c-Fos expression in CeA, PrL, and IL 30 min after the spontaneous recovery test.* (**a**) Photomicrograghs of representative images for c-Fos staining at 20X magnification in CeA, PrL and IL. (**b**) Expression of c-Fos protein after the spontaneous recovery test; values are mean$\pm$SEM number of c-Fos-positive cell counts per mm^2 in the different brain regions, n=6 per experimental condition, one-way ANOVA, $p>0.1$.

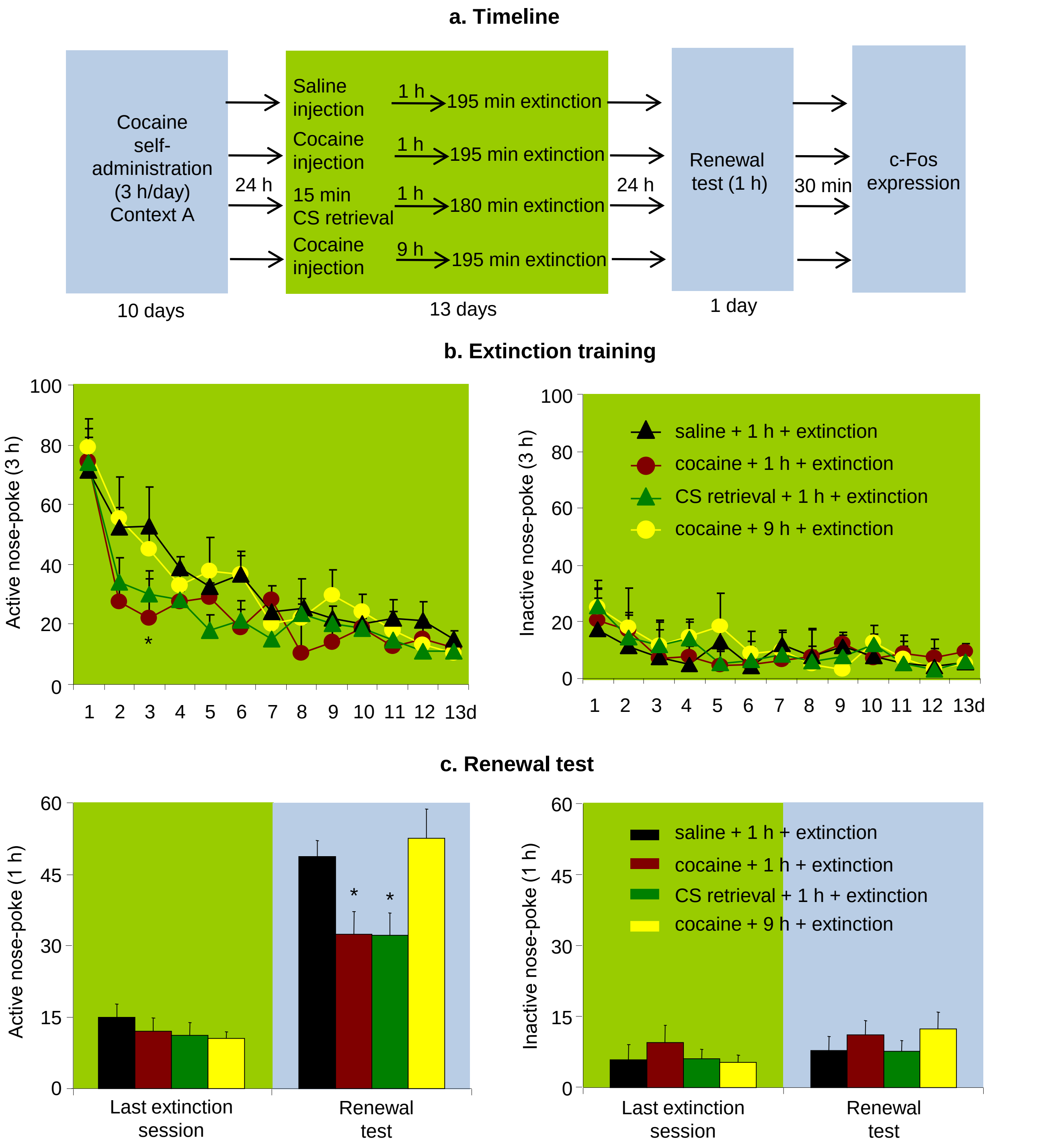

Supplementary Figure 5. *Exposure to the UCS and CS memory retrieval-extinction manipulations accelerated extinction responding and decreased renewal (context-induced reinstatement) of cocaine seeking.* (**a**) Timeline of the experimental procedure. (**b-c**) Mean ± SEM number of responses on the active and inactive nose-poke devices during the extinction phase and renewal test, n=9-10 per experimental condition. * Different from group “saline + 1 h + extinction”, mixed ANOVA**,** $p<0.05$.

a. Photomicrograghs of representative images for c-Fos staining

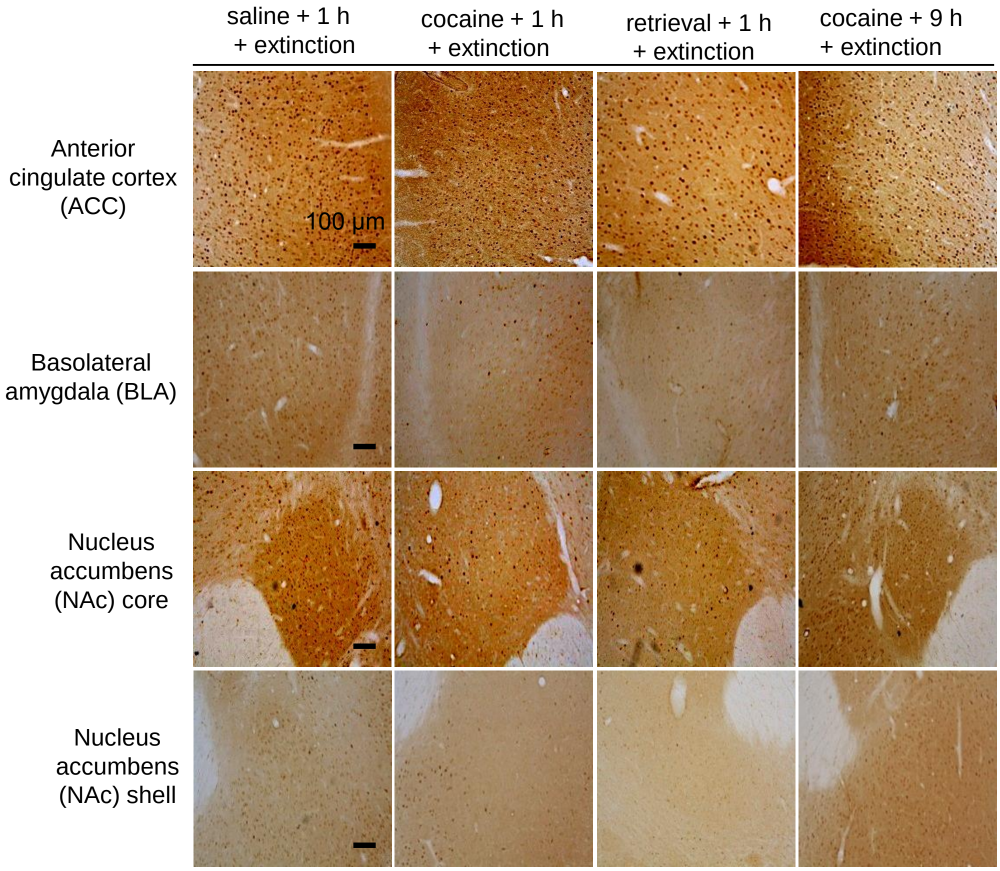

b. Number of c-Fos positive cells

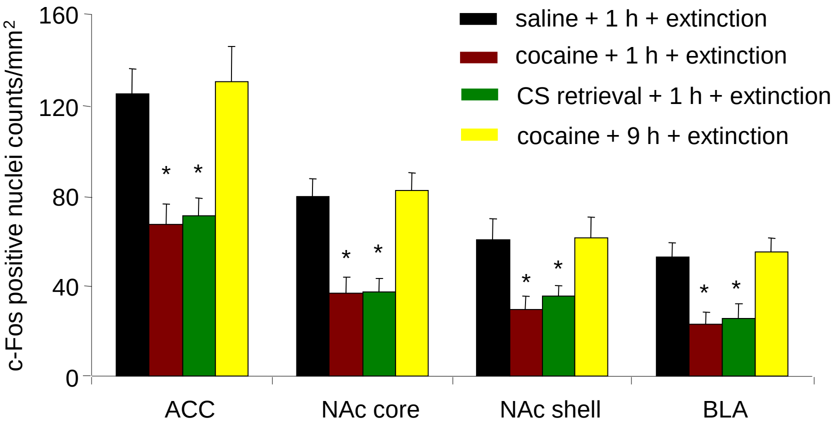

Supplementary Figure 6. *Exposure to the UCS and CS memory retrieval-extinction manipulations decreased c-Fos expression in ACC, NAc core, NAc shell and BLA 30 min after the renewal test.* (d) Photomicrograghs of representative images for c-Fos staining at 20X magnification in ACC, BLA, and NAc core and shell. (e) Expression of c-Fos protein after the renewal test; values are mean $\pm$ SEM number of c-Fos-positive cell counts per mm^2 in the different brain regions, n=6 per experimental condition. * Different from “saline + 1 h + extinction”, one-way ANOVA**,** $p<0.05$.

a. Photomicrograghs of representative images for c-Fos staining

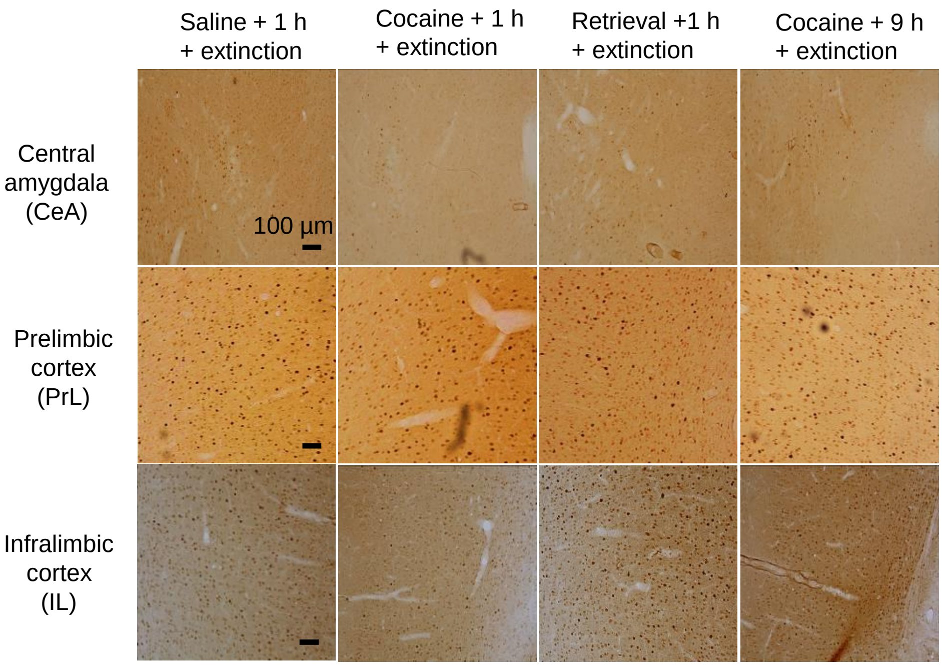

b. Number of c-Fos positive cells

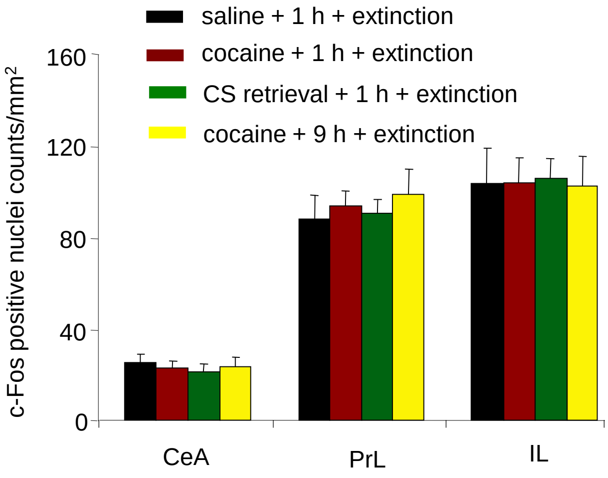

Supplementary Figure 7. *Exposure to the UCS and CS memory retrieval-extinction manipulations had no effect on c-Fos expression in CeA, PrL, and IL 30 min after the renewal test.* (**a**) Photomicrograghs of representative images for c-Fos staining at 20X magnification in CeA, PrL and IL. (**b**) Expression of c-Fos protein after the renewal test; values are mean ± SEM number of c-Fos-positive cell counts per mm^2 in the different brain regions, n=6 per experimental condition, one-way ANOVA, p>0.1.

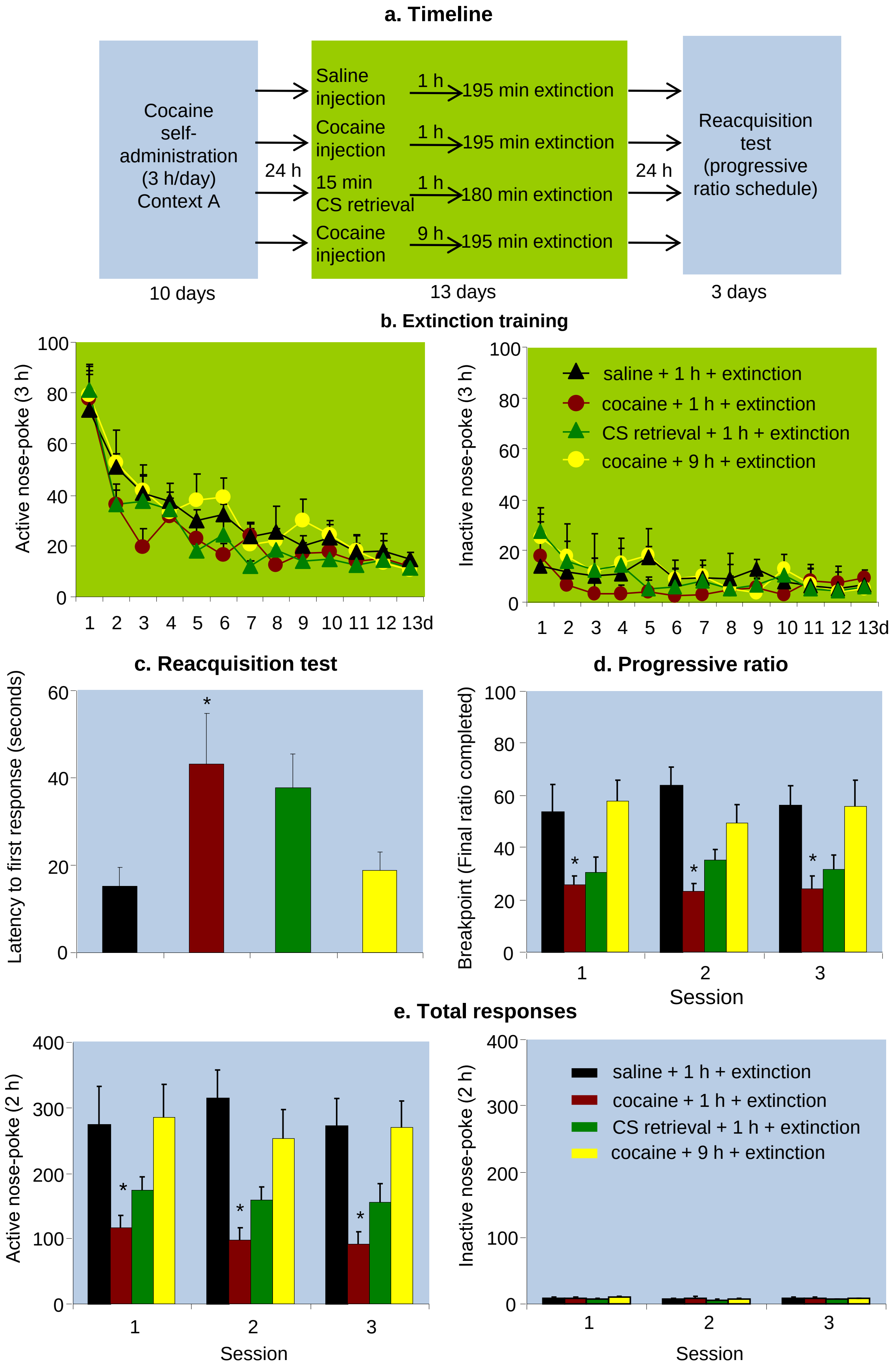

Supplementary Figure 8. *Exposure to the UCS memory retrieval-extinction manipulation decreased reacquisition of cocaine self-administration.* (**a**) Timeline of the experimental procedure. (**c**). Latency to the first nose-poke in the first reacquisition session. (**b,d,e**) Mean±SEM umber of responses on the active and inactive nose-poke devices during the extinction phase and the reacquisition test sessions, n=8-9 per experimental condition. * Different from group “saline + 1 h + extinction”, mixed ANOVA**,** p<0.05;.

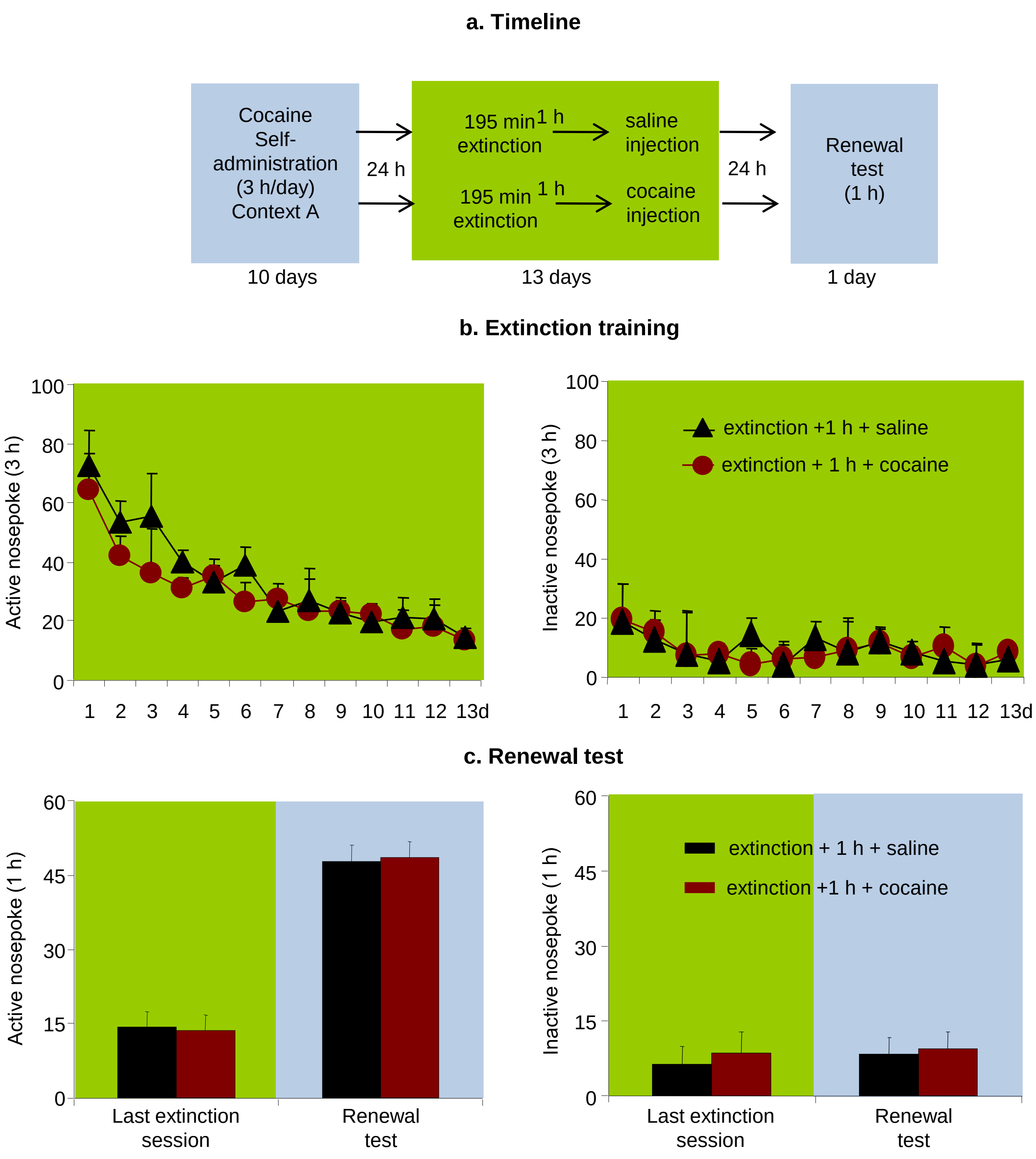

Supplementary Figure 9. *Reversal of the experimental conditions of the UCS memory retrieval-extinction manipulation (extinction followed by UCS retrieval) had no effect on renewal of cocaine seeking.* (**a**) Timeline of the experimental procedure. (**b-c**) Mean±SEM number of responses on the active and inactive nose-poke devices during the extinction phase and renewal test, n=8-9 per experimental condition, mixed ANOVA, p>0.1.

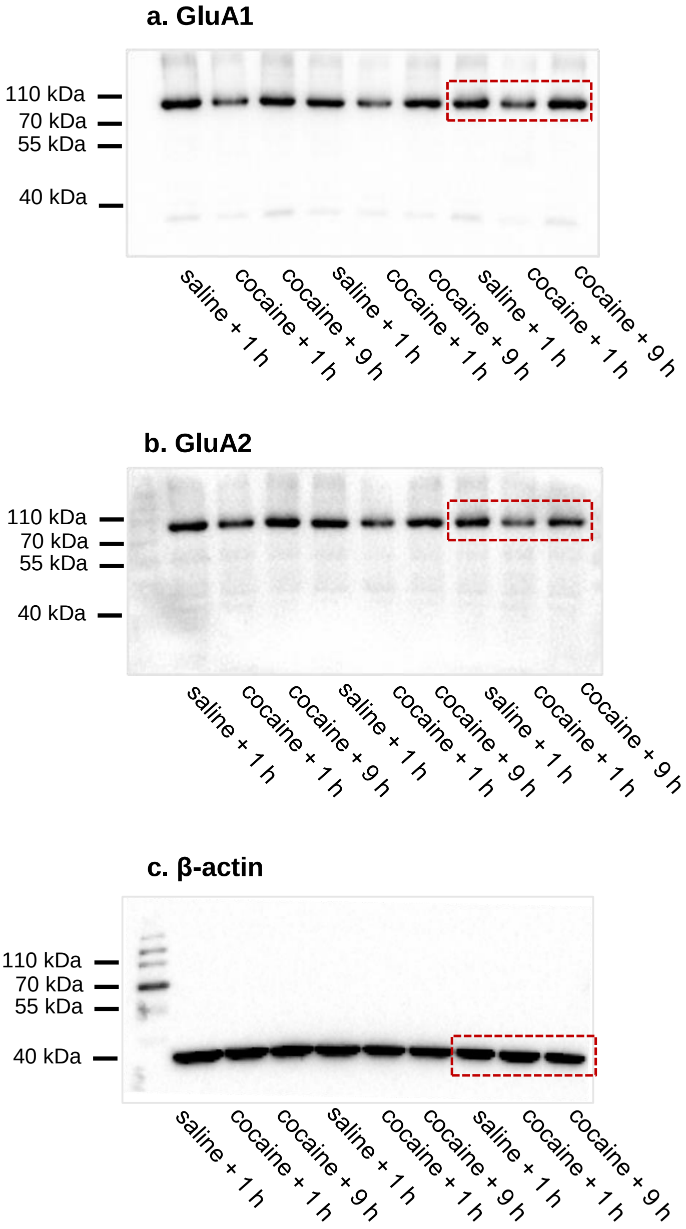

Supplementary Figure 10. *Full-length images of blots of GluA1 (**a**), GluA2 (**b**) and β-actin (**c**).* The quantification of the data is provided in Figure 5 in the main text.

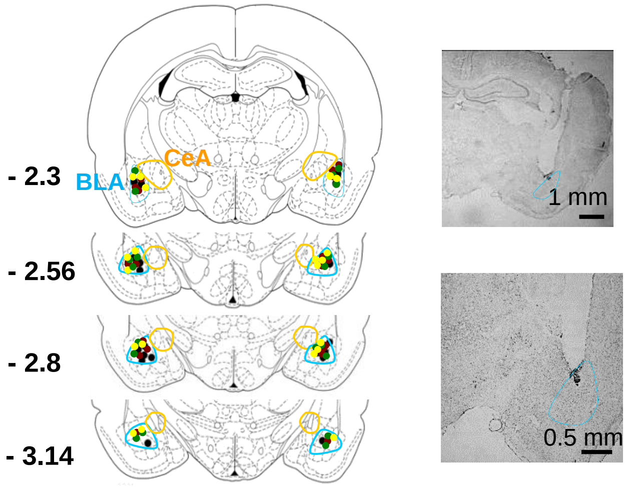

Supplementary Figure 11. *Schematic representation of injection sites in the basolateral amygdala for the 4 experimental groups.*
